# Investigation into the Molecular Mechanisms underlying the Anti-proliferative and Anti-tumorigenesis activities of Diosmetin against HCT-116 Human Colorectal Cancer

**DOI:** 10.1038/s41598-019-41685-1

**Published:** 2019-03-26

**Authors:** Sanaz Koosha, Zahurin Mohamed, Ajantha Sinniah, Mohammed A. Alshawsh

**Affiliations:** Department of Pharmacology, Faculty of Medicine, University of Malaya, 50603 Kuala Lumpur, Malaysia

## Abstract

Diosmetin (Dis) is a bioflavonoid with cytotoxicity properties against variety of cancer cells including hepatocarcinoma, breast and colorectal (CRC) cancer. The exact mechanism by which Dis acts against CRC however, still remains unclear, hence in this study, we investigated the possible molecular mechanisms of Dis in CRC cell line, HCT-116. Here, we monitored the viability of HCT-116 cells in the presence of Dis and investigated the underlying mechanism of Dis against HCT-116 cells at the gene and protein levels using NanoString and proteome profiler array technologies. Findings demonstrated that Dis exhibits greater cytotoxic effects towards HCT-116 CRC cells (IC50 = 3.58 ± 0.58 µg/ml) as compared to the normal colon CCD-841 cells (IC50 = 51.95 ± 0.11 µg/ml). Arrests of the cells in G2/M phase confirms the occurrence of mitotic disruption via Dis. Activation of apoptosis factors such as Fas and Bax at the gene and protein levels along with the release of Cytochrome C from mitochondria and cleavage of Caspase cascades indicate the presence of turbulence as a result of apoptosis induction in Dis-treated cells. Moreover, NF-ƙB translocation was inhibited in Dis-treated cells. Our results indicate that Dis can target HCT-116 cells through the mitotic disruption and apoptosis induction.

## Introduction

Colorectal cancer (CRC) is the third leading cause of cancer-related mortality worldwide. Alarmingly, 700,000 deaths were reported for CRC incidence in 2016^1^. It is expected that by 2030 the global rate of CRC will reach more than 2.2 million new cases and 1.1 million deaths^2^. Increase in incidence and mortality of CRC is different between developed and developing countries. Incidence and mortality rate of CRC in developed countries is higher, but there is trend of increasing incidence in countries with low and middle incomes^3^. Like other types of cancer cells, CRC cells have common hallmarks such as, uncontrollable growth, insensitivity to growth inhibitors, resistance to apoptosis, indefinite replicative potential and their angiogenesis ability, which helps tumors to survive and migrate to other parts of the body.

Besides surgery and radiotherapy, adjuvant chemotherapy drugs such as oxaliplatin and 5-fluorouracil (5-Fu) are commonly used for the treatment of CRC^4^. Despite frequent use of these chemotherapy drugs, there have been a large number of undesirable side effects observed during therapy, such as chest pain and cardiotoxicity^5^. Moreover, treatment with these drugs can lead to failure due to resistance of cancer cells^6^. Therefore, new therapeutic agents targeting different signaling pathways of cancer cells with lesser burden of side effects on normal cells are much desired. Therefore, natural products are considered as potential candidates due to their low side effects and high anti-cancer efficiency^7^.

Diosmetin (Dis) is a citrus flavonoid with anti-tumorigenesis properties against a variety of cancer cells including hepatocarcinoma, leukemia, breast, lung and prostate cancer^8^. Dis is able to inhibit the proliferation of cancer cells through different pathways. Although Dis inhibits polo-like kinase 1 (PLK1) as a progression factor during mitosis, proliferation of different cancer cells such as A549, MDA-MB 468, LNCaP and PC3 cells are inhibited in G0/G1 phase^8–12^. In prostate cancer, the arrest of cells occur as a consequence of reduction in protein expression of cyclin D, cdk 2 and 4. Moreover, reduction in protein expression of Bcl-2 and c-Myc, whilst overexpression of Bax, p27kip1 and Foxo3 encourages prostate cancer to progress to the apoptosis phase^8^. Dis induces apoptosis not only in prostate cancer but also in leukemia cells by activation of extrinsic apoptosis pathway and in hepatocarcinoma cells through the inhibition of NF-ƙB and activation of p53^12–14^. In addition, Dis was identified as a metastasis inhibitor in hepatocellular carcinoma (HCC) cells via reserve effect of this compound on matrix metalloproteinase MMP 2 and MMP 9^15^. Although the cytotoxicity of Dis against Colo205, HT-29 and Caco-2 colon cancer cells has been reported, the exact molecular mechanism of this compound in controlling proliferation is yet to be elucidated. In the present study, we investigated the anti-colorectal cancer effect of Dis against HCT-116 as one of the common human colorectal cancer cells. Besides, we introduce diosmetin (Dis) as a potent polyphenol for combating CRC as an alternative therapeutic agent. In addition, the molecular mechanisms involved and targeted signaling pathways were investigated at the gene and protein levels.

## Results

### Cytotoxic effect of diosmetin against colon cancer cells

**Figure 1:**
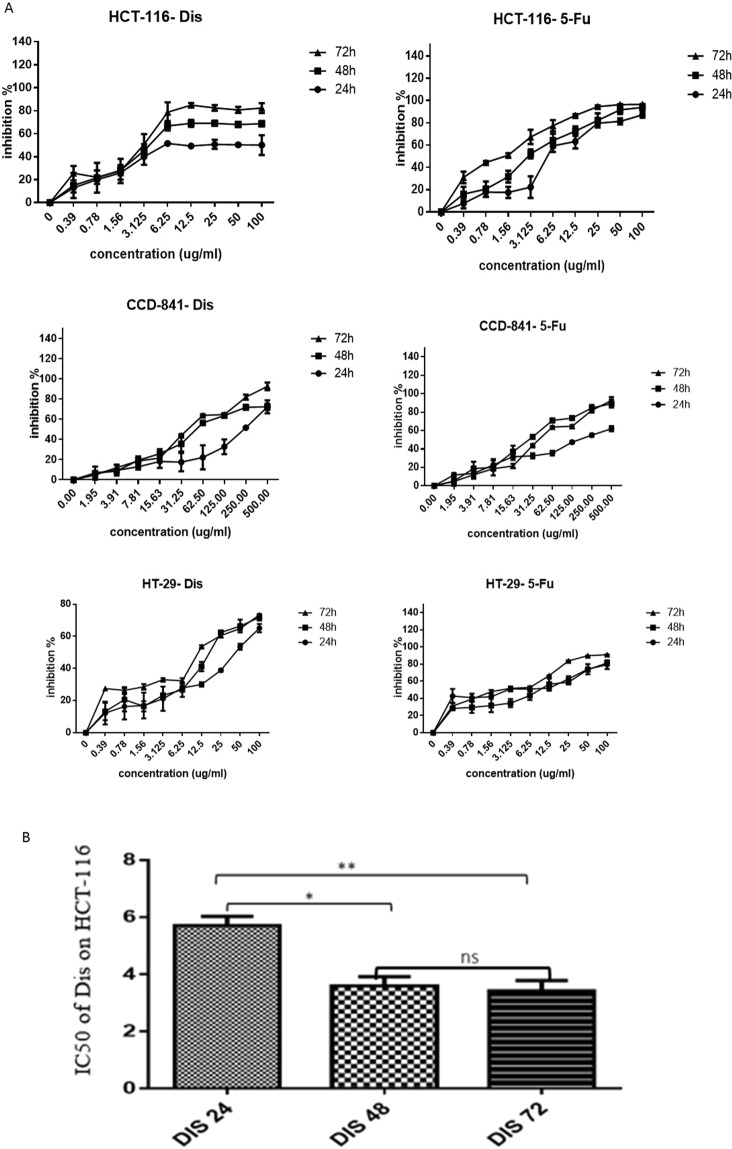
Cytotoxic effect of diosmetin (Dis) against HCT-116, HT-29 and CCD-841 cells. (**A**) Cells were treated with various concentrations of Dis or DMSO as vehicle control at different time points (24 h, 48 h and 72 h). Fluorouracil (5-Fu) was used as a positive control. (**B**) IC_50_ of Dis against HCT-116 cells after 48 h and 72 h of treatment were significantly lower compared to 24 h of treatment. All experiments were expressed in triplicates as mean ± SD. *p < 0.05, **p < 0.01, ns: no significant difference.

Cytotoxicity effect of Dis on HCT-116, HT-29 colon cancer cells, and CCD-841 normal colon cells were assessed using MTT assay. The IC_50_ of Dis against HCT-116 cells was 5.56 ± 0.48 µg/ml, 3.58 ± 0.58 µg/ml and 3.41 ± 0.64 µg/ml after 24 h, 48 h and 72 h, respectively. Moreover, the IC_50_ of Dis against HT-29 cells was 44.47 ± 5.31, 17.55 ± 2.96 and 11.46 ± 0.1, respectively at the same time points. However, CCD-841 treated cells with Dis showed IC_50_ of 236.46 ± 9.99 µg/ml, 51.95 ± 0.11 µg/ml and 40.51 ± 9.99 µg/ml, respectively at the same time points (Table 1). Among these three time points the IC_50_ of Dis treated HCT-116 after 48 h was significantly lower compared to the IC_50_ of Dis after 24 h while, IC_50_ of 48 h and 72 h was not significantly different. Therefore, the IC_50_ of 48 h was selected to investigate the underlying molecular mechanism and signaling pathways of Dis. Moreover, cytotoxicity of Dis was compared with 5-Fu as conventional anti-colon cancer drug (Table 1). Moreover, the IC_50_ of Dis against HT-29 was much higher as compared to HCT-116 at the same time points, therefore HCT-116 colon cancer cells was selected for further exploration of the mechanistic pathway of anti-cancer properties of Dis. Figure 1 shows the inhibitory percentage of Dis and 5-Fu against HCT-116, HT-29 and CCD-841 cells.

**Table 1:** Inhibitory effect of Dis against colon cancer and noraml colon cells.

Cell line	Dis		5-FU	
	IC_50_	SI	IC_50_	SI
**IC50 (μg/ml)/24** **h**				
HCT-116	5.56 ± 0.48	42.53	5.43 ± 0.48	36.27
CCD-841	236.46 ± 9.99		196.93 ± 14.74	
HT-29	44.47 ± 1.75	5.31	10.28 ± 2.7	19.15
**IC**_**50**_ **(µg/ml)/48** **h**				
HCT-116	3.58 ± 0.58	14.51	3.07 ± 0.34	9.23
CCD-841	51.95 ± 0.11		28.34 ± 0.67	
HT-29	17.55 ± 0.61	2.96	10.49 ± 1.3	2.7
**IC50 (µg/ml)/72** **h**				
HCT-116	3.41 ± 0.64	11.88	1.47 ± 0.4	9.85
CCD-841	40.51 ± 9.99		14.48 ± 0.68	
HT-29	11.46 ± 0.1	3.53	2.24 ± 0.53	6.46

HCT-116 and HT-29 colon cancer cells and CCD-841 non cancerous cells were treated with diosmetin (Dis), fluorouracil (5-Fu) for 24 h, 48 h and 72 h. Cell inhibitory was determined by 50% inhibition concentration (IC_50_). All results were expressed in three independent experiments with mean ± SD. Selectivity Index (SI) was calculated by dividing the IC_50_ of non cancerous (CCD-841) cells by the IC_50_ of colonc ancer (HCT-116 and HT-29) cells. SI is an index that gives an idea about the selectivity and the highest the value indicates more selective candidate.

### Diosmetin activates intrinsic and extrinsic pathways of apoptosis

**Figure 2:**
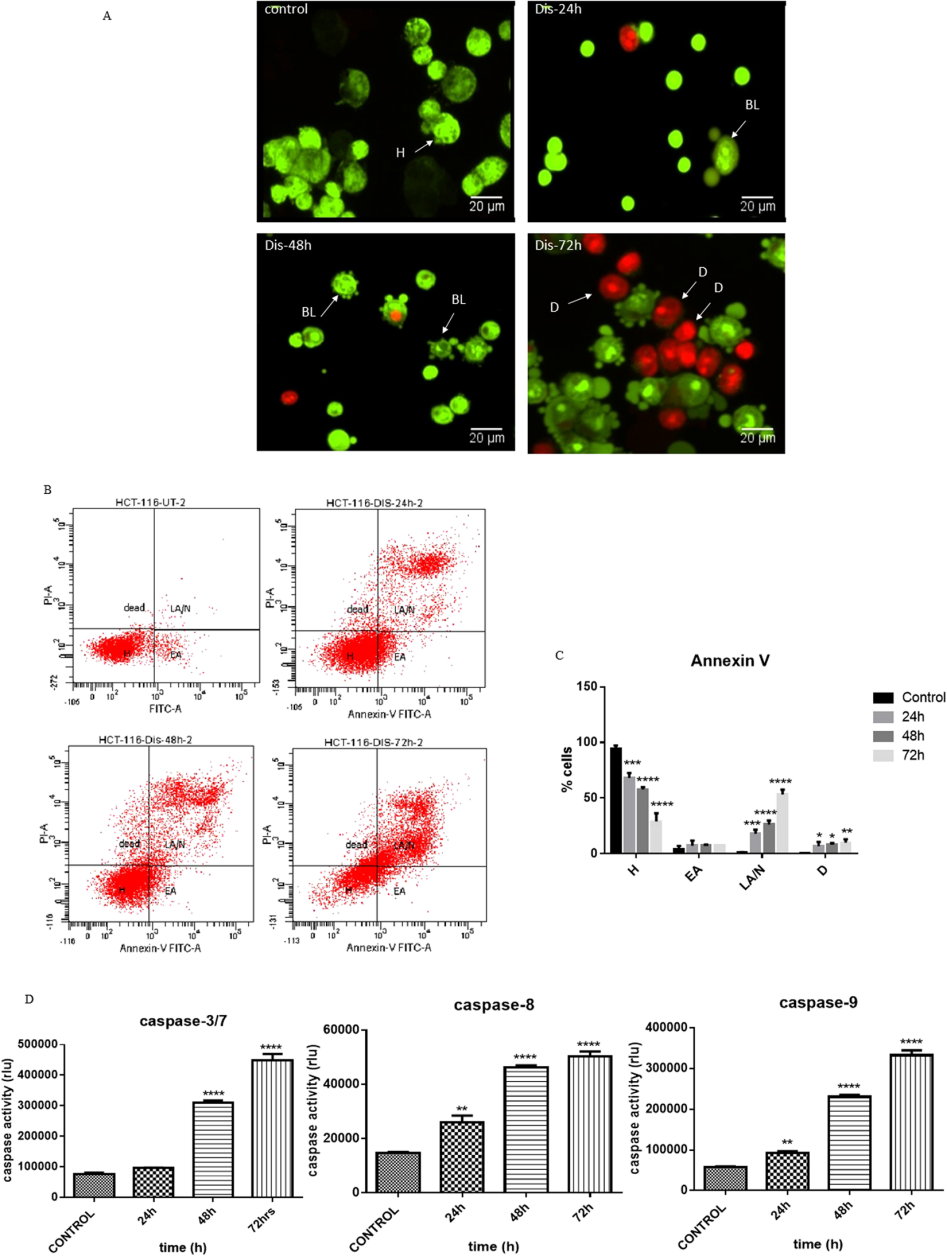
Induction of apoptosis in Dis treated HCT-116 cells. (**A**) Cells were treated with the IC_50_ of Dis and stained with AO/PI stain. Stained cells were introduced to florescent microscope. Dis changed the morphology of the cells with the passing of time. In the presence of Dis chromatin condensation and blebbing was observed. H: intact cells; BL: blebbing; D: dead (magnification x20). (**B**) Annexin-V vs. PI plots were generated via FACs technology. Cells were treated with Dis at different time points. Cells without any treatment were reserved as a control group. (**C**) The percentage of the cells in each stage. H: intact; EA: early apoptosis; LA/N: late apoptosis/necrosis; (**D**) dead. D. Luminescence analysis of caspases 3/7, 8 and 9 after 24 h, 48 h and 72 h treatment of HCT-116 cells with Dis. Data were expressed as mean ± standard deviation. *p < 0.05, **p < 0.01, ***p < 0.001, ****p < 0.0001 indicate significant differences compared to control.

As shown in Fig. 2A, AO/PI staining demonstrated that in the presence of Dis, morphology of HCT-116 cells was changed with the passing of time. After 48 h exposure of the HCT-116 cells to Dis, cells started to have appearance of blebbing, which is a sign for induction of apoptosis. After 72 h treatment, the numbers of dead cells increased. For Annexin-V assay, Fig. 2B shows that apoptosis in treated HCT-116 cells with Dis was induced in time dependent manner. In the control group, the population of cells in early apoptosis was increased from 4.15 ± 2.95% to 7.7 ± 0% after 72 h of treatment. Late apoptosis was observed (1.2 ± 0.3% control; 18 ± 3.6% after 24 h; 26.3 ± 3.6% after 48 h; and 53.15 ± 4.35% after 72 h) after treatment of the cells with Dis at the different time points (Fig. 2C). The obtained results demonstrated that Dis induced apoptosis in HCT-116 cells. In order to investigate which pathway of apoptosis was involved, the activity level of caspase 8 (extrinsic apoptosis pathway), caspase 9 (intrinsic apoptosis pathway) and caspase 3/7 (common in both apoptosis pathways) were measured using caspase cascade kit. As referred in Fig. 2D, in the presence of Dis, cleavage of caspase 3/7, 8 and 9 were significantly increased in a time dependent manner. These data shows that Dis instigates apoptosis through both the intrinsic and extrinsic pathways.

### Intrinsic pathway

**Figure 3:**
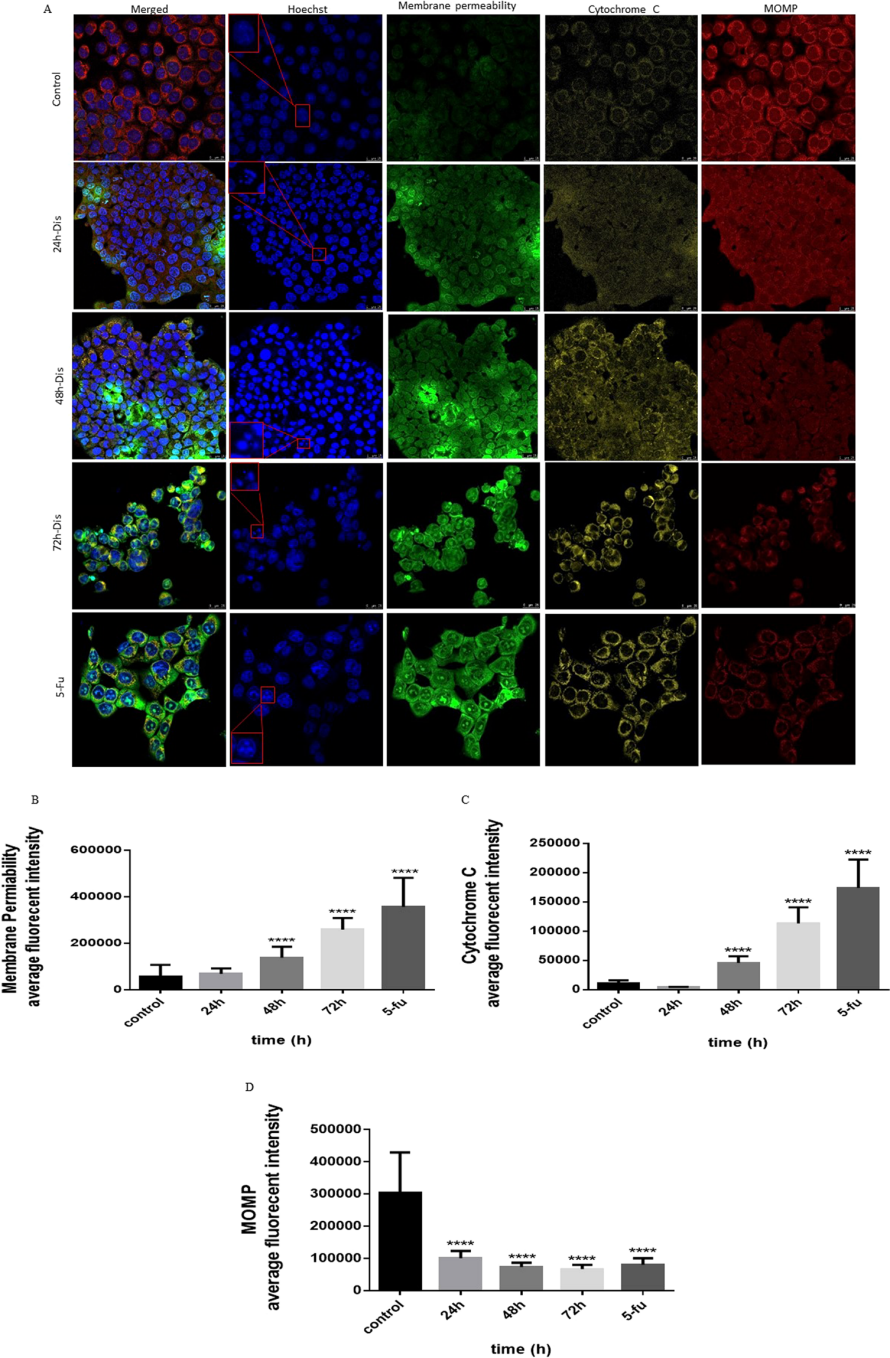
Effect of Dis on induction of intrinsic apoptotic pathway. (**A**) Cells were treated with Dis (3.58 µg/ml) at different time points and stained with Hoechst 33342 (blue), YoYo dye (green), cytochrome C (yellow) and mitotracker (red) dyes. Dis caused increases in cell permeability (**B**), releases of Cytochrome C from mitochondria to cytosol (**C**), and losses of the mitochondrial membrane potential as shown by the decreases in Mitotracker uptake (**D**). MOMP: mitochondria outer membrane permeabilization. Data were expressed as mean ± standard deviation. ****p < 0.0001 indicates significant difference compared to control.

**Figure 4:**
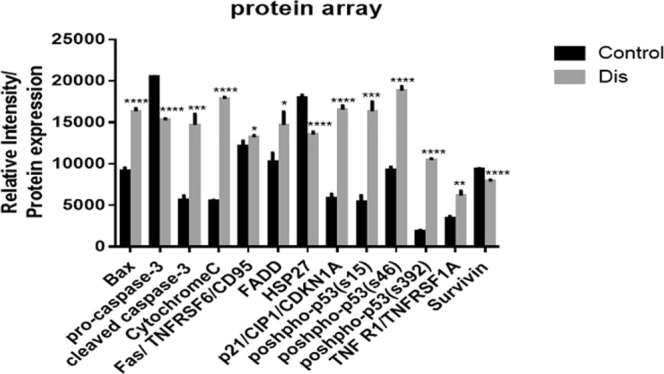
Proteome profiling of apoptosis associated proteins. Relative level of apoptotic proteins in the cell lysate of untreated and Dis treated cells. Cells were treated with 3.58 µg/ml of Dis for 48 h. Apoptotic protein levels were determined using proteome profiler array kit. Data were expressed as mean ± standard deviation. *p < 0.05, **p < 0.01, ***p < 0.001, ****p < 0.0001 indicate significant difference compared to control.

After evaluating the intrinsic and extrinsic apoptotic pathways, the features of each pathway were assessed in detail for Dis treated cells. HCT-116 cells showed cell permeability aggravation and loss of mitochondrial membrane potential when exposed to Dis as compared to the untreated cells. As shown in Fig. 3, nucleus Hoechst 33342 (blue) is intact and uniform in untreated HCT-116 cells while treated cells have fragmented nucleus. In the absence of Dis, YoYo dye (green) is not able to penetrate inside the cells since the cell membrane is unified. Therefore, in control group intensity of green dye is low compared to the treated cells. On the other hand, release of Cytochrome C (yellow) from mitochondria was observed in Dis-treated cells but not in the untreated cells. In the treated group, reduction of Mitotracker dye (red) in the cytosol indicates mitochondrial damage and increase in mitochondrial outer membrane permeabilization (MOMP) compared to the control group. Moreover, gene expression results indicate that expression of *bax* which is a key factor in intrinsic apoptosis pathway, significantly increased in the presence of Dis^16^ (Table 2). The protein expression level of Bax and Cytochrome C were accelerated while protein expression of Survivin (caspase 9 inhibitor) significantly decreased in treated cells compared to the control. On the other hand, Hsp27 (Cytochrome C inhibitor) expression significantly decreased in both gene and protein levels (Fig. 4 & Table 2).

**Table 2:** Expression levels of extrinsic and intrinsic apoptotic genes in Dis treated cells.

Gene symbol	Log2 fold change	Lower confidence limit	Upper confidence limit	P-value	FDR
*hdac5*	−2.6	−4.08	−1.14	0.00838	0.0394
*hspb1 (hsp27)*	−2.2	−3.11	−1.25	0.00178	0.0142
*hdac2*	−0.6	−0.724	−0.508	3.75E-06	0.000279
*sin3*	−0.5	−0.765	−0.234	0.00612	0.0311
*hdac1*	−0.5	−0.685	−0.287	0.00139	0.0127
*bax*	0.5	0.27	0.655	0.00153	0.0134
*setd2*	0.7	0.335	1.01	0.00449	0.026
*tnfrsf10b*	0.8	0.364	1.18	0.00598	0.0307
*fas*	0.8	0.204	1.47	0.0319	0.0976
*cdkn1a (p21)*	1.5	1.27	1.66	4.11E-07	0.000184

### Extrinsic pathway

In addition to activate Caspase 8, dead receptors such as TNFR and Fas showed increase in their activities in the gene and protein levels. Consequently expression of FADD as a dead domain protein was significantly increased (Fig. 4). Apart from the significant role of protein and genes which stimulated intrinsic and extrinsic pathways individually, the high level protein expression of some other factors such as phosphorylation of P53 plays a significant role on both pathways simultaneously in treated cells. Phosphorylation of P53 (s15 and s46) can induce P21 which is known as a cell proliferation inhibitor^17^. As shown in Table 2, gene expression of *setd2* as an inducer of P53 significantly increased, while the expression of inhibitors of P53 (*hdac1*, *2*, *5* and *sin3*) were decreased significantly (*P* < 0.05)^18–21^. Protein expression of pro-caspase 3 was significantly reduced in Dis treated group as compared to the control group (fold change = 0.75). On the other hand, protein expression of caspase 3 cleavage was increased significantly up to 2.61 fold change in Dis treated group compared to the control group (Fig. 4).

### Dis inhibits the translocation of NFƙB

**Figure 5:**
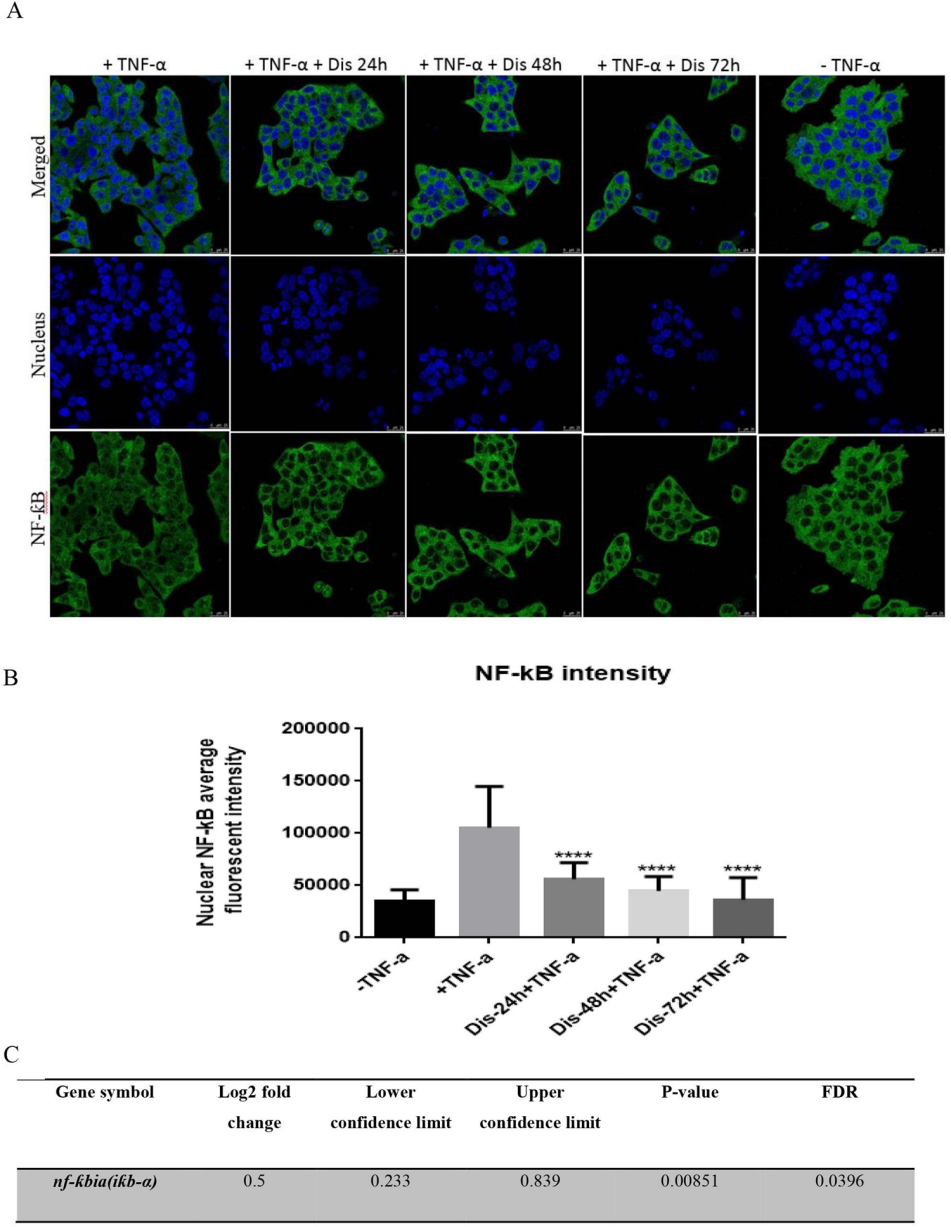
Dis inhibits NF-ƙB translocation. (**A**) Dis exposed cells were stimulated with TNF-α. Translocation of NF-ƙB was detected in the presence and absence of Dis. (**B**) Bar chart indicates the amount of translocated TNF-α to the nucleus. Data in triplicates were expressed as mean ± standard deviation. ****p < 0.0001 indicates significant difference compared to control (+TNF-α). C. Activation of NF-ƙB inhibitor (*iƙb-α*) gene in the presence of Dis.

Although activation of TNF receptor can induce the extrinsic pathway, it can also inhibit apoptosis through translocation of NF-ƙB from cytoplasm to nucleus. In order to understand if Dis can inhibit NF-ƙB translocation, NF-ƙB assay was performed. As demonstrated in Fig. 5A and B, following treatment of the cells with Dis, translocation of NF-ƙB was significantly reduced as compared to the untreated group. Moreover, as shown in Fig. 5C Dis elevates the gene expression of NF-ƙB inhibitor (*iƙb-α*)^22^.

### Dis inhibits cell proliferation and arrests cells in G2/M

**Figure 6:**
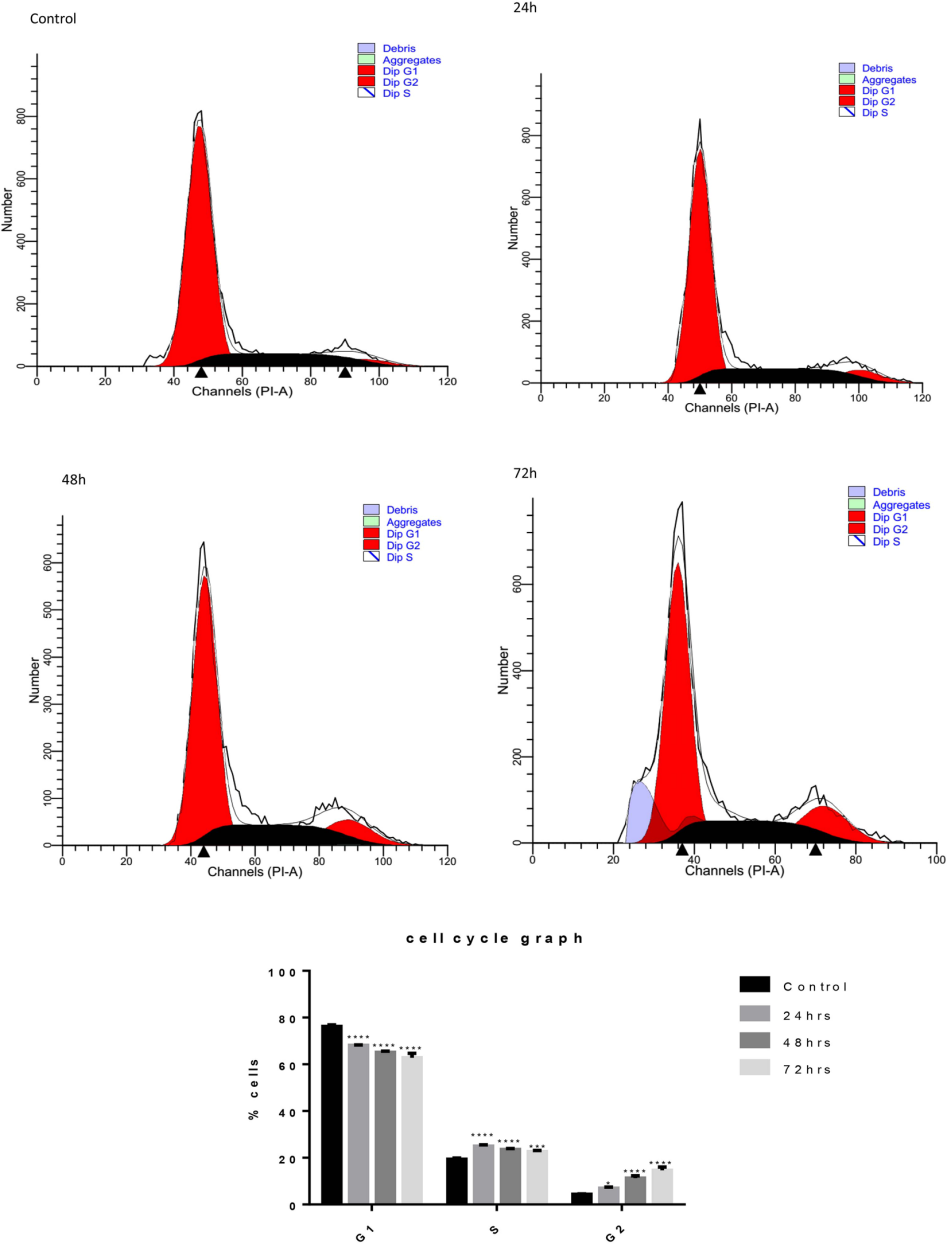
Dis inhabits cell proliferation through G2/M arrest. (**A**) Cell proliferation of HCT-116 was performed in the absence and presence of Dis at various time points (24 h, 48 h and 72 h) using flow cytometry. Figure demonstrates that cells were arrested in G2/M phase. (**B**) Quantitative analysis demonstrated that cells were arrested in G2/M phase. Data were expressed as mean ± standard deviation. *p < 0.5, ***p < 0.001, ****p < 0.0001 indicate significant differences compared to control.

Flow cytometer was used to evaluate the effect of Dis on cell arrest by measuring the DNA content of the cells in the presence of Dis at different time points as compared to untreated cells. As shown in Fig. 6A,B, the first peak represents the DNA content of the cells in G0/G1 and the second peak represents the DNA content of the cells in G2/M. Accumulation of the DNA content in G2/M with time indicates that the cells were arrested during the mitotic division.

In order to identify the mechanism underlying mitotic arrest, signaling pathway involved in G2/M arrest was investigated using Nanostring. As shown in Table 3, in the presence of Dis, expression of the genes that are involved in mitosis division (*ttk*, *pttg2*, *mad2l2*, *stmn1*, *smc3* and *rad21*) were significantly reduced^23–31^. Meanwhile, gene expression of *cyclin A* and *B* inducer such as *cdc25* was significantly decreased, expression of the *cyclin A* and *B* inhibitors (*p21*, *gadd45* and *stratifin*) were accelerated and consequently, gene expression of *cyclin A* and *B* which are known to be cell regulators in G2/M, were inhibited^32–39^.

**Table 3:** Expression levels of cell cycle associated genes in Dis treated cells.

Gene symbol	Log2 fold change	Lower confidence limit	Upper confidence limit	P-value	FDR
*hdac5*	−2.6	−4.08	−1.14	0.00838	0.0394
*id2*	−2.0	−2.27	−1.64	1.98E-06	0.000217
*stmn1*	−1.5	−1.83	−1.11	4.35E-05	0.00139
*rbx1*	−1.2	−1.96	−0.52	0.00972	0.0439
*ttk*	−1.0	−1.22	−0.741	4.28E-05	0.00139
*pttg2*	−0.9	−1.24	−0.508	0.00162	0.0135
*rad21*	−0.8	−0.942	−0.591	2.66E-05	0.00108
*smc3*	−0.7	−0.963	−0.352	0.00291	0.0188
*ccna2 (cyclin A)*	−0.6	−0.88	−0.408	0.000693	0.00775
*hdac2*	−0.6	−0.724	−0.508	3.75E-06	0.000279
*mad2l2*	−0.6	−0.816	−0.395	0.000486	0.00624
*cdc25*	−0.6	−0.84	−0.289	0.00387	0.023
*sin3*	−0.5	−0.765	−0.234	0.00612	0.0311
*hdac1*	−0.5	−0.685	−0.287	0.00139	0.0127
*ccnb1 (cyclin B)*	−0.5	−0.772	−0.177	0.0142	0.0565
*Fubp1*	−0.5	−0.732	−0.372	0.000318	0.00547
*id1*	−0.5	−0.805	−0.26	0.005	0.0276
*sfn (stratifin)*	0.6	0.416	0.823	0.000339	0.00561
*gadd45*	0.8	0.578	0.933	3.24E-05	0.00121
*cdkn1a (p21)*	1.5	1.27	1.66	4.11E-07	0.000184

### Potential role of Dis in DNA synthesis

Proliferation of Dis-treated cells is not only influenced by mitosis slippage, but also by inhibition of DNA replication. The results illustrated that the expression of genes, which are involved in DNA repair (*c19orf40 (faap24)*, *ubb*, *nbn* and *msh2*), DNA packaging (*hist1h3h*, *hist1h3b*, *h3f3a* and *h2a*) and DNA replication (*pole2*, *hells*, *pcna*, *mcm2*, *4*, *7*, *cdc7*and *prkar2b*) were significantly decreased (Table 4). Therefore, the obtained results suggest a potential role of Dis in inhibiting DNA synthesis in HCT-116 treated cells.

**Table 4:** Expression levels of DNA synthesis (DNA replication and DNA repair) associated genes in Dis treated cells.

Gene symbol	Log2 fold change	Lower confidence limit	Upper confidence limit	P-value	FDR
*Prkar2b*	−1.6	−1.98	−1.28	1.66E-05	0.000742
*hist1h3h*	−1.5	−1.81	−1.19	1.22E-05	0.000683
*pole2*	−1.4	−1.81	−1.03	0.000102	0.00242
*hist1h3b*	−1.2	−1.48	−0.964	1.48E-05	0.000735
*c19orf40 (faap24)*	−1.2	−1.62	−0.801	0.000401	0.00608
*h3f3a (h2a)*	−0.9	−1.03	−0.692	8.76E-06	0.000559
*h2afx*	−0.9	−1.17	−0.683	7.42E-05	0.00195
*ubb*	−0.9	−1.21	−0.59	0.000457	0.00624
*cdc7*	−0.8	−1.13	−0.455	0.00173	0.014
*msh2*	−0.7	−0.878	−0.424	0.000495	0.00624
*mcm2*	−0.6	−0.86	−0.272	0.00545	0.0284
*mcm4*	−0.6	−0.843	−0.331	0.00202	0.0146
*mcm7*	−0.6	−0.777	−0.374	0.000517	0.00624
*pcna*	−0.6	−0.917	−0.325	0.00337	0.0206
*hells*	−0.6	−0.954	−0.303	0.00531	0.0283
*nbn*	−0.5	−0.74	−0.238	0.00508	0.0277
*cdkn1a (p21)*	1.5	1.27	1.66	4.11E-07	0.000184

### BMP is a potential mechanism involved in Dis triggered apoptosis

Nano string results indicate that the gene expression of *bmp8A*, *smad 9*, *id 1* and *2* in Dis-treated HCT-116 cells were significantly decreased (*p* < 0.05) (Table 5). Gene *bmp* belongs to the *tgf beta* superfamily which stimulates *smads* including *smad 9*. Activation of *smad 9* can regulate the transcriptional factors such as *id1* and *2* which are known as cell proliferative factors and *p21* inhibitors^40–42^. Therefore in the presence of Dis, apoptosis activation and cell cycle inhibition may be triggered though the inhibition of TGF-β signaling pathway.

**Table 5:** Expression levels of BMP pathway associated genes in Dis treated cells. BMP pathway factors were inhibited in Dis treated cells.

Gene symbol	Log2 fold change	Lower confidence limit	Upper confidence limit	P-value	FDR
*smad9*	−2.7	−3.8	−1.58	0.00142	0.0127
*bmp8a*	−2.2	−3.17	−1.25	0.002	0.0146
*id1*	−0.5	−0.805	−0.26	0.005	0.0276
*id2*	−2.0	−2.27	−1.64	1.98E-06	0.000217

## Discussion

Apoptosis and cell proliferation inhibition are considered to be the main targets for chemotherapeutic drugs in colon cancer treatments^43,44^. Besides the side effect of these drugs, treated tumors may develop resistance to these chemotherapeutic agents and prolong the proliferation^5^. Contrary to chemotherapeutic drugs, anti-cancer drugs from natural sources have shown lesser side effects. These limitations encourage new researches to focus on developing more potent therapeutic drugs from natural sources^45,46^. Several natural products have been reported to possess anti-tumorigenesis properties including quercetin, kaempferol, baicalein and apigenin^7^. Dis is a flavonoid which is known for its anti-inflammatory and antioxidant properties^10^. Based on previous studies Dis has anti-proliferative, anti-metastasis and pro-apoptotic effect on breast cancer and hepatocellular carcinoma cells^10,13,15^. Moreover, cytotoxic effect of Dis against Colo205, HT-29 and Caco-2 cells demonstrates that Dis has anti tumorigenesis properties against human colorectal cancer^10,47,48^. However the exact molecular mechanisms underlying the anti-colon cancer effects of Dis are not well established. In the present study, we demonstrated that Dis inhibits cell proliferation and activates apoptosis signaling pathways in human colorectal cancer. Moreover, our study clearly demonstrates that activation of apoptosis and proliferation inhibition is likely to be intensified through the inhibition of BMP pathway and NF-ƙB.

Our results indicate that Dis was cytotoxic for HCT-116 colon cancer cells (IC_50_ = 3.58 ± 0.58 µg/ml) however the cytotoxicity of this compound on non-cancerous colon cells was low at the same time point (IC_50_ = 51.95 ± 0.11 µg/ml). Therefore, Dis might be considered as a potent candidate to treat colon cancer. The present study not only demonstrates the cytotoxicity of Dis against colon cancer but also shed a light on the possible molecular signaling pathways of this compound against HCT-116 cells. Inhibitory effect of Dis against HCT-116 was assessed in different time points. IC_50_ of Dis at 48 h (3.58 ± 0.58 µg/ml) was significantly lower as compared to the IC_50_ of this compound after 24 h (5.56 ± 0.48 µg/ml). However, no significant difference was observed between the IC_50_ of Dis at 48 h and 72 h (3.41 ± 0.64 µg/ml). The IC_50_ of Dis after 48 h was slightly higher than the IC_50_ of 5-Fu (3.07 ± 0.34 µg/ml) as a registered drug at the same time point. Despite the similarity in the IC_50_ doses of these two drugs, there was a significant difference between the IC_50_ of Dis (51.95 ± 0.11 µg/ml) and 5-Fu (28.34 ± 0.67 µg/ml) on normal colon cells CCD-841 after 48 h. Therefore, Dis might be a safer agent with lesser cytotoxic effect on normal cells as compared to 5-Fu. Cytotoxicity properties of Dis in previous studies demonstrates that Dis has the lowest effect on HCC (IC_50_ = 50 µg/ml) followed by Colo205 (IC_50_ = 29 µg/ml) and HepG-2 (IC_50_ = 12 µg/ml) after 24 h treatment. Moreover, IC_50_ of Dis against non-cancerous cells such as MCF-10 (IC_50_ = 30 µg/ml) and SK-Hep-1 (IC_50_ = 100 µg/ml) were higher compared to the cancer cells at the same time point^10,13,15,47^. By comparing cytotoxicity results of HCT-116 cells with the other cell lines in previous studies, it seems that HCT-116 cells (5.56 ± 0.48 µg/ml) has more sensitivity to Dis compared to the other types of cell lines except MDA-MB 468 (IC_50_ = 1.35 µg/ml) after 24 h treatment. Therefore it can be concluded that Dis has potent anti-tumor activity with less cytotoxic effect on normal cells. The possible reason for the extreme difference in the dosage of Dis towards colon cancer cells and normal colon cells, may lie in stimulation of apoptosis pathways by Dis and interference with mitotic progression in cancer cells.

**Figure 7:**
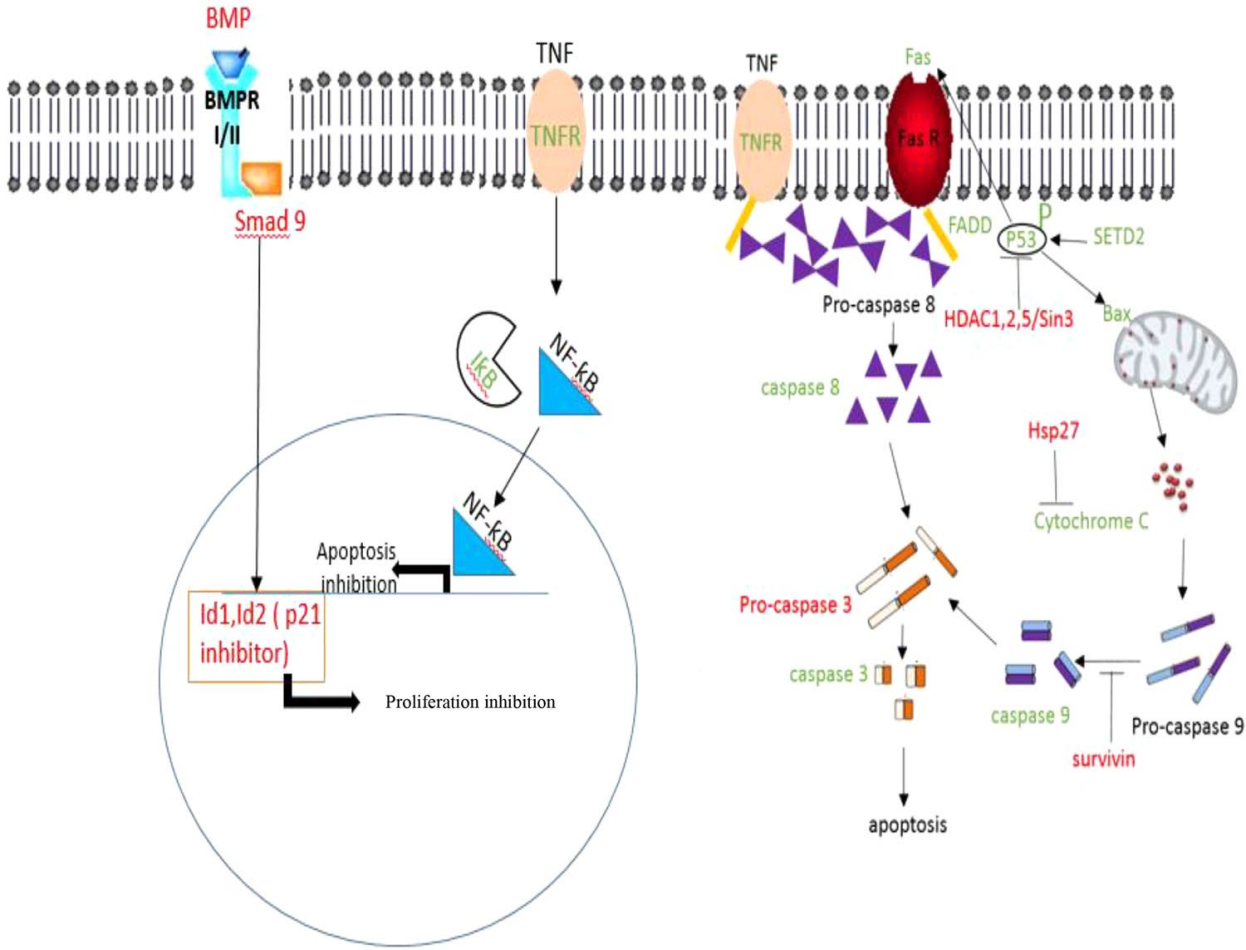
Illustration summarizes the molecular mechanism and signaling pathways underlying the effect of Dis on HCT-116 cells. Dis triggers intrinsic and extrinsic apoptotic pathway. Dis induces P53 phosphorylation, which is upstream of Bax. Bax increases the permeability of mitochondria, hence Cytochrome C leaks into the cytoplasm. Release of Cytochrome C promotes the cleavage of pro-Caspase 9 into an active form. Activation of Caspase 9 leads to the activation of other caspase members such as Caspase 3/7. *hdacs/sin3*, Hsp27 and Survivin which are known as inhibitors of P53, Cytochrome C and Caspase 9, respectively were down-regulated in the presence of Dis. When Fas and TNF-α were activated by P53, it activates Pro-Caspase 8, which is attached to the death domain (FADD) of Fas and TNF receptors. This attachment triggers the cleavage and activation of Caspase 8, which activates the caspase cascades. In the presence of Dis, NF-ƙB is arrested by *iƙb* (NF-ƙB inhibitor), hence the translocation of NF-ƙB to nucleus was inhibited. Dis is able to inhibit the *p21* inhibitors through the inhibition of *bmp*. In the presence of Dis, *bmp* is not able to change the assembly of *smad9*. Therefore, smad9 is not translocated to the nucleus, hence activation of *id1* and *id2* were inhibited. Green: up regulated, Red: down regulated, inhibition:–|, induction: →.

Eternal life of cancer cells occurs when their apoptotic signaling pathways are not activated. Apoptotic agents such as Dis can promote the destiny of cancer cells towards death by activating apoptotic factors such as caspase cascades. Activation of Caspase 8 and 9 occurs as a result of activation of the extrinsic and intrinsic apoptosis pathway, respectively. Meanwhile activation of Caspase 3/7 is involved in both apoptotic pathways. As shown in Fig. 7, activation of intrinsic apoptotic pathway is dependent on the stimuli from inside the cells. Apoptotic mediators accumulate in the intermediate space between the two membranes of mitochondria. Therefore, pro-apoptotic factors such as Bax increase the permeability of mitochondria through mitochondria outer membrane permeabilization (MOMP) process. As a result, the release of apoptotic mediators such as Cytochrome C combines with pro-Caspase 9 to form a complex named apoptosome. When pro-Caspase 9 cleaves to Capspase 9 it activates its downstream caspase cascades such as Caspase 3 and 7. Inhibitor proteins such as Survivin which is a member of inhibitors of apoptosis proteins (IAP) can inhibit the activation of Caspase 9 and 7 by blocking their active sites^16^.

In the present study, activation of Caspase 9 in Dis-treated cells together with increase in mitochondrial membrane permeability, release of Cytochrome C and Bax indicate that Dis can induce the intrinsic apoptotic pathway. Moreover, in the presence of Dis, protein expression of IAPs such as Survivin and Hsp27 (Cytochrome C inhibitor) is reduced in cancer cells.

In addition to intrinsic apoptosis pathway, extrinsic apoptosis pathway is triggered by death factors such as Fas and TNF-α. Caspase 8 acts as a key factor in this pathway^16^. In our study, HCT-116 treated cells with Dis increase the Fas and TNF receptor at both gene and protein levels. When the complex of death ligands and receptors in Dis-treated cells increase, configuration of the receptors will change and adaptor proteins such as FADD bind to the cytoplasmic end of the receptors. Increase in the expression level of FADD in treated cells cause the activation of Caspase 8 which initiates a cascade of Caspase activation such as Caspase 3 and 7^16^. Rapid ignition of apoptosis in Dis-treated cells, is partially related to the activation of P53 protein (Fig. 7). P53 is one of the most important tumor suppressors, which is altered in most human cancer cells. P53 is able to induce apoptosis by up regulation of death receptors such as Fas and pro-apoptotic Bcl-2 family member Bax^49,50^. Our gene expression results illustrated that expression of *setd2* as an inducer of *p53* was increased whereas expression level of *p53* inhibitors such as *hdac1*,*2*,*5* and *sin3* was decreased^18–21^. Although gene expression of *p53* inducers and inhibitors were altered in Dis treated cells, gene expression of *p53* remains unchanged. Our protein array results illustrate that activation of P53 occurred as a results of phosphorylation of P53 in ser15, ser46 and ser392^51,52^.

NF-ƙB is a transcription factor, which is a key mediator in inflammatory response. NF-ƙB is activated through inflammatory factors such as TNF-α. Besides the inflammation, NF-ƙB has other downstream effectors that contribute to tumorigenesis and apoptosis inhibition. In general, translocation of NF-ƙB from cytoplasm to the nucleus causes apoptosis inhibition via activation of IAPs such as Survivin. Moreover, NF-ƙB can be accumulated in cytoplasm by NF-ƙB inhibitor (*iƙb-α*)^22,53^. In Dis-treated cells, TNF-α receptors were increased, which in turn activates the extrinsic apoptosis pathway. As described previously, TNF-α is an NF-ƙB inducer. In our study, despite elevation of TNF-α receptors in treated cells with Dis, translocation of NF-ƙB was inhibited via overexpression of *iƙb-α*. Moreover, reduction in protein expression of Survivin confirms NF-ƙB inhibition. Hence, Dis activates the apoptosis both through the engagement of apoptotic factors and inhibition of NF-ƙB (Fig. 7).

Generally, bone morphogenetic protein (BMP) that belongs to TGF-β family is upregulated in CRC resulting in activation of BMP signaling pathway. BMP is known to be a key factor in tumorigenesis^54^. Binding of BMP to their receptors change the assembly of BMP-specific receptors named Smads. The rearranged Smads translocate to the nucleus and activate DNA binding inhibitors (Id1 and Id2), which are known as P21 and cell proliferation inhibitors^41,42,55,56^. Moreover elevation in gene expression of *id1* is known to be a marker for human colon cancer^57^. Previous studies indicate that apoptosis is induced by inhibition of TGF-β pathway in Dis treated HepG2 cells^13^. In our study, HCT-116 treated cells with Dis show reduction in gene expression of *bmp8a (smad 9)*, *id1* and *2*. Therefore, overexpression of *p21* might be accomplished as a result of reduction in expression of *bmp* signaling pathway members (Fig. 7). As a result, BMP pathway can be considered as a potential target pathway for Dis against HCT-116.

Dis not only activates apoptosis in colon cancer cells, but also inhibits cancer cells proliferation. Cell cycle encoded genes that are highly mutated in cancer cells, which lead to uncontrollable proliferation. Therefore, therapeutic drugs that target cell cycle component such as cyclin inhibitors are considered to be important drug strategies. Therefore, proteins affecting the function of the mitotic spindle are considered to be another important target for anti-colon cancer drugs^58–60^. Any disruption in mitosis components by anti-cancer drugs can result in mitosis slippage and G2/M arrest. In the present study, Dis inhibits cell cycle and arrest cancer cells in G2/M phase. Moreover, key genes that are involved in mitosis have been interrupted in Dis-treated cells.

Cohesion complex which has been constructed by *rad21*, *smc3*, *smc1a* and *stag1/2* hold the sister chromatids together during the G2 phase and mitosis until metaphase to anaphase transition^23^. Based on the study in 2011, highly expressed Rad21enhances the resistance of breast cancer to 5-Fu^24^. Moreover, it was established that Smc3 is elevated in CRC^25^.

In mitosis, *stmn1* gene plays an important role in the regulation of microtubule dynamic by promoting microtubule depolymerisation. The gene *stmn1* is highly expressed in a variety of cancer cells and it is associated with poor prognosis^26^.

The gene *mad2l2 (rev7)* is another component of the mitotic spindle assembly checkpoint that prevents the onset of anaphase until all chromosomes are properly aligned at the metaphase. In colorectal cancer, *mad2l2* is highly expressed^27^. In another study on ovarian cancer, suppression of *mad2l2* increases cisplatin sensitivity^28^.

The high expression of *pttg2* is required for normal tubulin distribution. Abnormal distribution of cytoskeletal proteins occurs in *pttg2*-depleted cells, thus reduction of *pttg2* expression resulted in rounded morphology and cells die by P53- and P21-dependent apoptosis^29^.

In the absence of *ttk (mps1)* gene, cell proliferation is significantly attenuated and apoptosis rate is significantly increased^30^. As described by Ling *et al*., there is a correlation between high levels of *ttk* with tumor grades in CRC^31^. The gene *ttk* is involved in chromosome alignment and spindle pole assembly during the mitosis but the most permanent function of *ttk* is to ensure that the sister chromatids are attached to the spindle at kinetochore.

**Figure 8:**
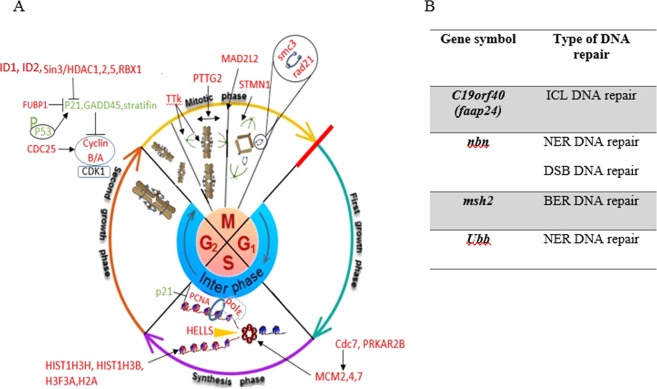
Illustration of cell cycle inhibition in presence of Dis in HCT-116 cells. (**A**) In Dis treated cells, mitosis were disrupted due to the down-regulation of mitosis associated genes. Reduction in *rad 21* and *smc3* gene expression inhibits the formation of cohesion complex in G2 phase therefore sister chromatids cannot hold together. Separation of sister chromatids during metaphase were disrupted when *pttg2* and *ttk* gene expressions were inhibited. Regulation of microtubule dynamic was altered when *stmn1* was inhibited in Dis treated cells. Moreover, in the presence of Dis, *cyclin A/B* and cyclin A/B inducer (*cdc25*) were inhibited whereas their inhibitors (*p21*, *gadd45* and *stratifin*) were overexpressed. Gene expression of p21 inhibitors (*id1*, *id2*,*sin3/hdac1*,*2*, *5*, *rbx1* and *fubp1*) were reduced in the presence of Dis. In cells, *mcms* forms the replication fork while *hells* unwind the dsDNA and *pole* extends the leading strand. Dis disrupts the DNA replication fork, ssDNA generation and extension of leading strand. DNA synthesis accelerator was inhibited in the presence of Dis due to inhibition of *pcna*. Gene *p21* was induced as an activator of *pcna*. Moreover, Dis inhibits the inducers of *mcms* such as *cdc7* and *prkar2b*. Dis inhibits gene expression of *hist1h3h*, *hist1h3b(h3)*, *h3f3a* and *h2a*, which are involved in nucleosome packaging. (**B**) List of different DNA repair genes. In the presence of Dis, DNA repair genes were inhibited. Green: up regulated, Red: down regulated, inhibition:–|, induction: →.

As it described in Fig. 8A, mitotic spindle assembly was disrupted in Dis-treated cells through the inhibition of *rad21*, *smc3*, *stmn1*, *mad2l2 (rev7)*, *pttg2* and *ttk (mps1)* genes. Therefore, massive mitosis disruption occurs due to the infirmed sister chromatid attachment, interruption of microtubule dynamic, mitosis phase interference and chromosome attachment.

On the other hand, *cyclin A* and *B* which are responsible for regulation and coordination of cells passage through G2/M and S phases were inhibited in the presence of Dis. Degradation of *cyclin B* is essential for mitosis slippage. In the case of disruption in any of the mitotic components, *cyclin B* degradation is prevented until errors are corrected^32^. However, when mitotic checkpoints are not capable of correcting errors and microtubule disruption is massive, the cell will then exit mitosis through mitotic slippage by degrading *cyclin B*^33^.

Mitotic slippage on one hand and inhibition of *cdc25* (*cyclin B* inducer) and overexpression of *cyclin B* inhibitors such as *p21*, *gadd45* and *stratifin* on the other, attenuated *cyclin A and B*^34–37^. In addition, Dis inhibits the *id1*, *id2*, *fubp*, *rbx1 (roc1)* and *hdacs/sin3* complex which is considered as *p21* inhibitors. The gene *rbx1* is one of the requirements for cancer survival and in the absence of this gene, cells enter apoptosis and cell arrest in G2/M phase^38,39^. As aforementioned, P53 is activated in Dis-treated cells. P53 does not only regulate apoptosis signaling pathway, but also arrest the cells by inducing P21^49^. Thus, it can be concluded that Dis persuades HCT-116 cells to undergo mitotic slippage and cell arrest in G2/M phase.

As described before, previous studies claimed that Dis has anti-proliferative properties by arresting MDA-MB 468 breast cancer cells in G1 phase^10^. By comparing these data with ours, it can be concluded that the molecular mechanism of Dis seems to vary in different type of cells.

Proliferation rate in cancer cells are higher as compared to the normal cells. For this matter, DNA replication needs to be accelerated. This feature makes cancer cells more susceptible to chemotherapeutics drugs which targets DNA replication and repair^61^.

The *mcm2*, *4* and *7* are involved in forming replication fork and recruitment of other DNA replication related elements. They are also responsible for activation of Helicase which unwinds dsDNA to generate ssDNA templates for DNA polymerase function^62,63^. Subunits of *msm*s are mainly induced through *cdc7* and *prkar2b* genes^64,65^. While DNA is unwound, DNA polymerase is initiated DNA replication. During the replication of DNA, DNA pol ε extend the leading strand and *pcna* accelerates the DNA synthesis. DNA pol ε is not only involved in replication, but it has role in DNA repair^66,67^. The function of *pcna* is inhibited by *p21*^68^. In the presence of Dis, gene expression of *mcm2*,*4*,*7*, *hells* (Helicase), *cdc7*, *prkar2b*, *pole2* (DNA pol ε) and *pcna* were inhibited while, *p21* expression was elevated in HCT-116 cells (Fig. 8A).

During and after DNA replication, based on the type of error, DNA repair system initiates repair of DNA. Any disruption in DNA repair system, causes accumulation of damaged DNA. Since DNA repair system is defective in cancer cells, finding a way to turn dysregulated repair system against themselves and induce tumor death, is a goal for DNA repair inhibitor agents^69^. In our study, genes such as *msh2*, *c19orf40 (faap24)*, *nbn* and *ubb*, which are involved in different types of DNA repair system were inhibited in Dis-treated cells (Fig. 8B)^70–74^.

Beside the inhibition of genes which are involved in DNA synthesis and DNA repair by intermediation of Dis, expression of those genes which are involved in forming nucleosome such as *hist1h3h*, *hist1h3b(h3)*, *h3f3a* and *h2a* were decreased^75^. In order to construct chromosomes, DNA needs to condense through different form of packaging. Formation of nucleosome is the basic unit for chromosome construction in which segment of DNA wound around eight histone proteins including *h3* and *h2a*^75,76^. As described, Dis is able to inhibit HCT-116 proliferation not only through mitotic slippage, but also by the interruption of DNA synthesis system, inhibition of DNA repair system and delay in forming nucleosome (Fig. 8A).

Our data indicates the anti-cancer properties of Dis against HCT-116 cells. Mechanism of action of Dis against HCT-116 might be through activation of apoptosis, mitotic slippage and NF-ƙB inhibition. Besides, the other potential anti-tumorigenesis mechanism of Dis might be through inhibition of DNA synthesis/ repair system and BMP pathways. Our data support previous studies for apoptotic properties of Dis through activation of P53, Bax and inhibition of NF-ƙB. However, in spite of prostate cancer and breast cancer, cell cycle proliferation inhibited in G2/M phase for HCT-116 cells through mitotic slippage and inhibition of *cylin A/B* rather than in G0/G1 phase^8–14^.

Despite previous results which claim that Dis inhibits TGF-β and Smad 3 in HepG-2, no significant difference were observed in HCT-116 treated cells^13^. However, our results indicate that Dis inhibit *bmp* and *smad 9* in HCT-116 cells. Moreover, our results did not support previous findings based on inhibition of *mmp 2* and *mmp 9* in HCC cells^15^. These differences indicate that mechanism of action of Dis might vary between different types of cancer cells.

## Conclusion

In conclusion, our findings demonstrate that Dis has anti-cancer properties against HCT-116 cells and less cytotoxic effect against non-cancerous colon cells. Our study claims that the mechanism of action of Dis against HCT-116 cancer cells might be through the induction of apoptosis and inhibition of cell proliferation. Dis is able to induce apoptosis mediated by the mitochondria and apoptosis mediated by membrane death receptor. Dis activates the intrinsic apoptotic pathway through a mechanism in which Dis induces Bax to form a channel in the outer membrane of mitochondria and releases Cytochrome C to activate Caspase 9, whereas Caspase 8 is activated when dead domain of TNF-α/Fas cleaves pro-Caspase 8. Dis also accelerates apoptosis when NF-ƙB arrested by IƙB and hence transcription of anti-apoptotic factors is inhibited. Induction of pro-apoptosis component in the presence of Dis on one hand and inhibition of anti-apoptosis on the other, accelerate apoptosis trend in HCT-116 cells.

Dis arrests HCT-116 cells in G2/M phase either through inhibition of *cyclin A/B* or mitotic slippage. Activation of *cyclin A/B* inhibitors (*p21*, *gadd45*, *stratifin)* and prevention of their inducer (*cdc25*) play an important role in the down regulation of *cyclin A/B* in Dis-treated cells. Moreover, in the presence of Dis, mitosis is disrupted in HCT-116 cells due to early detachment of sister chromatids and interruption in microtubule dynamic. In addition, by inhibition of *bmp*, cell cycle arrest is accelerated due to activation of *p21*. As BMP and DNA synthesis/ repair inhibition are considered as potential pathways that are affected in Dis-treated cells, which need further investigations to explore the exact mechanisms by which diosmetin affected these pathways.

## Methodology

### Compound and cell lines

Diosmetin was purchased from Abcam (Cambridge, UK) with the molecular weight of 300.26 g/mol and purity >98%. 5-Fluorouracil (5-FU) was obtained from MP biomedical, lllkirch, France. HCT-116, HT-29 (human colon cancer) and CCD-841 (human non-cancerous) cell lines were obtained from ATCC, USA. All assays were performed in three independent experiments and the results are presented as mean ± SD of triplicates.

### Cell culture

HCT-116, HT-29 (colon cancer) and CCD-841 (non-cancerous) cell lines were obtained from ATCC (VA, USA). Cells were cultured in DMEM medium (HyClone, Logan, Utah, USA) supplemented with 10% FBS and 1% penicillin and streptomycin (Biowest, France). Cells were incubated in incubator at 37 °C with 5% CO_2_. Negative control for all assays was represented by 0.02% DMSO (vehicle) in untreated medium.

### Cytotoxicity assay

Cytotoxicity property of Dis against HCT-116 and CCD-841 cells were measured by MTT assay as described by Mossman^77^. Briefly, cells were treated with different concentration of Dis and 5-Fu (positive control) with serial dilution factor of 2 for 8 concentrations in 96 well-plate for different time points (24 h, 48 h and 72 h). Subsequently at the end of each time point 20 µl of MTT (5 mg/ml, Affymetrix,Cleveland, OH, USA) were added to each well followed by 4 h incubation. The solutions were aspirated after 4 h and 100 µl of DMSO were added to each well. Absorbance for each well was read at 570 nm as measured by using microplate reader (Tecan Infinite multimode, Männedorf, Switzerland). Cytotoxicity (cell death) was measured by using the following formula:

$$\mathrm{Cell}\,\mathrm{death}\,( \% )=\frac{\mathrm{sample}\,\mathrm{OD}\,-\,\mathrm{control}\,\mathrm{OD}}{\mathrm{control}\,\mathrm{OD}}100$$

This assay was performed in triplicates in three independent experiments. The cytotoxicity property of Dis was presented as IC_50_ value.

### Acridine orange/propidium iodide (AO/PI) double staining assay

Morphological changes of HCT-116 cells in the absence and presence of Dis were assessed by AO/PI (Santa cruz, CA, USA) double staining assay as previously described^78^. In brief, cells were seeded in 25 cm^2^ culture flask followed by treatment with Dis at different time points of 24 h, 48 h and 72 h. Cells were then harvested and washed with PBS. Pellets were stained with 10 µl of AO/PI. Morphological changes of the cells were detected using the fluorescence-inverted microscope (Nikon, Japan) in which green color represents healthy cells while dead cells were represented by red color.

### Annexin-V-FITC assay

Inductions of early and late stages of apoptosis by Dis were investigated using Annexin-V-FITC assay according to the manufacturer’s instructions (BD Biosciences, San Jose, CA, USA). Briefly, 10^6^ HCT-116 cells were seeded in 25 cm^2^ flask followed by treatment with Dis for 24 h, 48 h and 72 h. Treated cells were harvested and washed twice with cold PBS and suspended in binding buffer followed by transferred of 10^5^ cells to the 6 ml polystyrene round-bottom FACS tubes. Cells were incubated with 5 µl of PI and 5 µl of Annexin-V-FITC and introduced to the FACSCanto™II instrument (BD, San Jose, CA, USA).

### Bioluminescent assays for caspase 8, 9 and 3/7 activities

To assess the effect of Dis on caspase cascades in treated cells, activities of caspase-Glo 3/7, caspase-Glo 8 and caspase-Glo 9 assays (Promega, Madison, WI, USA) were performed. Briefly, HCT-116 cells with the density of 10^4^ cells/well were seeded in 96 well-plates. Cells were treated with Dis for 24 h, 48 h and 72 h. This assay was performed in triplicates for each time point. A total of 6 wells were preserved for samples without cells but with media and reagents as the blank samples, and for the untreated cells as negative control. At the end of incubation time, 100 µl caspase-glo mixture were added to each well and incubated for 1 h in the dark according to the manufacturer’s instructions. Optical density was measured via multimode microplate reader (Tecan Infinite, Männedorf, Switzerland).

### Multiple cytotoxicity assay

Cellomics multiparameter cytotoxicity 3 kit (Thermo Scientific™, Pittsburgh, PA, USA) was used to investigate the vital apoptotic events in HCT-116 cells in the presence of Dis. Cytotoxicity 3 assay was used to provide information about the changes in nuclear morphology, cell membrane integrity, cytochrome C release and mitochondrial outer membrane permeabilization (MOMP). HCT-116 cells were seeded in 24 well-plate and covered by a cover slide followed by treatment with Dis for 24 h, 48 h and 72 h. Treated cells were stained with YoYo dye (life technology, CA, USA) and Mitotracker dye (life technology, CA, USA) followed by fixation and blocking procedures according to the manufacture’s protocol. Primary cytochrome C (Thermo Scientific™, Pittsburgh, PA, USA) antibody and secondary DyLight 650 (Thermo Scientific™, Pittsburgh, PA, USA) were added to each well and incubated for 1 h in the dark. Hoechst 33342 dye (Thermo Scientific™, Pittsburgh, PA, USA) was added in the last step to stain the nuclei of the cells. Cells were washed and cover slides were transferred on the slide and introduced to confocal Leica TCS SP5 II microscope (Leica Microsystems, Mannheim, Germany). Intensity of each dye was measured by using LAS X software.

### Translocation of NF-κB

Confocal microscope was used to detect the suppressive effect of Dis on translocation of NF-κB from cytoplasm to nucleus induced by TNF-α (Santa Cruz, CA, USA) using cellomics nucleus factor-κB (NF-κB) activation kit (Thermo Scientific™, Pittsburgh, PA, USA). HCT-116 cells were seeded and treated with Dis at different time points in 24 well-plate covered with cover slide. An amount of 150 ng/ml of TNF-α was used as a stimulator for 24 h. Cells were then fixed and stained according to the manufacturer’s instructions. Translocation of NF-κB was investigated via confocal Leica TCS SP5 II microscope (Leica Microsystems, Mannheim, Germany) and intensity of NF-κB was measured by using LAS X software.

### Cell cycle

Flow cytometry analysis was performed to determine any disruption during the cell cycle of HCT-116 cells in the presence of Dis. HCT-116at density of 2 × 10^6^ cells were seeded and treated for 24 h, 48 h and 72 h in a 75 cm^2^ flask. Negative control was treated with 0.02% DMSO. After fixation of the cells with 70% ethanol, cells were preserved in −20 °C overnight followed by washing and staining with 500 µl of PI/Rnase dye (BD Biosciences, San Jose, CA, USA) and incubated for 30 min. DNA content analysis was performed using BD FACSCanto II flow cytometer instrument and BD FACS Diva software (BD FACSCanto™II, San Jose, CA, USA). A total of 15,000 events per sample were recorded for analysis. ModFit LT version 3.0 software was used for analyzing the data.

### Gene expression profiling

Gene expressions of the cancer canonical pathways were measured by NanoString nCounter. HCT-116 cells were treated with Dis for 48 h and total RNA were extracted by using RNeasy Mini Kit (QiAgen, Hilden, Germany) according to the manufacture’s protocol. Concentrations of RNA were checked through NanoDrop 2000C (Thermo Scientific™, Pittsburgh, PA, USA). Pancancer pathways panel on Nanostring nCounter system (Nanostring Technologies, Inc., Seattle, WA, USA) were used to investigate the gene expression profile of cancer pathways. A total of 770 genes were investigated and compared between Dis treated and untreated cells. The list of investigated genes is provided as supplementary data (Table S1). RNA was hybridized, purified and immobilized before being aligned on the nCounter cartridge and introduced to nCounter.

Digital Analyzer was utilized for data collection according to the manufacturer’s instruction. Briefly, RNA samples were added to the master mix contain hybridization buffer and reporter CodeSet. Capture ProbeSet was added to this mixture and introduced to pre-heated 65 °C thermal cycler for 16 h incubation. After removing the unbound probe, target/probe were immobilized and aligned on the nCounter Cartridge. nCounter digital analyzer was used to collect the nCounter cartridge data and data were analyzed using nSolver 3.0 analysis software. Significant genes were choose based on the false discovery rate (FDR) <0.05 and log2 fold change ≥  ± 0.5 supplementary data (Figure S1).

### Protein array

Apoptosis protein expression profiling was performed using human apoptosis array (R&D Systems, Minneapolis, MN, USA) according to the manufacturer’s protocol. In brief, 2 × 10^6^ HCT-116 cells were seeded and treated with Dis in 75 cm^2^ flask and incubated for 48 h. Cells were harvested and lysed via lysis buffer reagent and was quantified using Pierce BCA protein assay kit (Thermo Scientific™, Pittsburgh, PA, USA). The membrane provided was blocked in blocking solution reagent and treated overnight with cell lysates protein. The membrane was washed and subsequently incubated with detection antibody cocktail and streptavidin horseradish peroxidase-conjugated. Chemiluminescent reagents were added to the membrane and then exposed to X-ray film to detect the protein expression levels in treated cells as compared to untreated cells. Images were quantified via ImageJ software (NIH, USA). The list of investigated proteins is provided as supplementary data (Table S2).

### Statistical analysis

All the data were expressed as mean ± standard deviation (SD) of three replicates. Statistical analyses were performed using one-way analysis of variance (ANOVA) with Tukey’s multiple comparisons. Data were analyzed with graph pad prism, version 6.07 for windows. The values of *p* < 0.05 were considered significant.

## Supplementary information

Dataset

## Data Availability

List of investigated genes and proteins are available in supplementary data. Gene expression of investigated genes are presented in volcano plot in supplementary data.
