# Supplementary material for: Investigation into the Molecular Mechanisms underlying the Anti-proliferative and Anti-tumorigenesis activities of Diosmetin against HCT-116 Human Colorectal Cancer
Source: Sci Rep. 2019 Mar 26;9:5148. doi: 10.1038/s41598-019-41685-1 (PMC6435658; doi:10.1038/s41598-019-41685-1)

Investigation into the Molecular Mechanisms underlying the Anti-proliferative and Anti-tumorigenesis activities of Diosmetin against HCT-116 Human Colorectal Cancer

Sanaz Koosha, Zahurin Mohamed, Ajantha Sinniah, Mohammed A. Alshawsh*

Department of Pharmacology, Faculty of Medicine, University of Malaya, 50603 Kuala Lumpur, Malaysia.

* Corresponding Author e-mail: alshaweshmam@um.edu.my

Sanaz Koosha: Department of Pharmacology, Faculty of Medicine, University of Malaya, 50603 Kuala Lumpur, Malaysia, e-mail: nazkoosha@gmail.com

Zahurin Mohamed: Department of Pharmacology, Faculty of Medicine, University of Malaya, 50603 Kuala Lumpur, Malaysia, e-mail: zahurin@um.edu.my

Ajantha Sinniah: Department of Pharmacology, Faculty of Medicine, University of Malaya, 50603 Kuala Lumpur, Malaysia, e-mail: ajantha.sinniah@um.edu.my

Mohammed A. Alshawsh: Department of Pharmacology, Faculty of Medicine, University of Malaya, 50603 Kuala Lumpur, Malaysia, e-mail: alshaweshmam@um.edu.my

Supplementary data

Table S1: List of investigated genes

Official Symbol	Accession	Official Full Name
ABL1	NM_005157.3	c-abl oncogene 1, non-receptor tyrosine kinase
ACVR1B	NM_004302.3	activin A receptor, type IB
ACVR1C	NM_145259.2	activin A receptor, type IC
ACVR2A	NM_001616.3	activin A receptor, type IIA
AKT1	NM_005163.2	v-akt murine thymoma viral oncogene homolog 1
AKT2	NM_001626.2	v-akt murine thymoma viral oncogene homolog 2
AKT3	NM_181690.1	v-akt murine thymoma viral oncogene homolog 3
ALK	NM_004304.3	anaplastic lymphoma receptor tyrosine kinase
ALKBH2	NM_001001655.2	alkB, alkylation repair homolog 2 (E. coli)
ALKBH3	NM_139178.3	alkB, alkylation repair homolog 3 (E. coli)
AMER1	NM_152424.3	APC membrane recruitment protein 1
AMH	NM_000479.3	anti-Mullerian hormone
ANGPT1	NM_001146.3	angiopoietin 1
APC	NM_000038.3	adenomatous polyposis coli
APH1B	NM_001145646.1	APH1B gamma secretase subunit
AR	NM_001011645.1	androgen receptor
ARID1A	NM_006015.4	AT rich interactive domain 1A (SWI-like)
ARID1B	NM_020732.3	AT rich interactive domain 1B (SWI1-like)
ARID2	NM_152641.2	AT rich interactive domain 2 (ARID, RFX-like)
ARNT2	NM_014862.3	aryl-hydrocarbon receptor nuclear translocator 2
ASXL1	NM_001164603.1	additional sex combs like 1 (Drosophila)
ATM	NM_138292.3	ataxia telangiectasia mutated
ATR	NM_001184.2	ataxia telangiectasia and Rad3 related
ATRX	NM_000489.3	alpha thalassemia/mental retardation syndrome X-linked
AXIN1	NM_181050.1	axin 1
AXIN2	NM_004655.3	axin 2
B2M	NM_004048.2	beta-2-microglobulin
BAD	NM_004322.3	BCL2-associated agonist of cell death
BAIAP3	NM_003933.4	BAI1-associated protein 3
BAMBI	NM_012342.2	BMP and activin membrane-bound inhibitor
BAP1	NM_004656.2	BRCA1 associated protein-1 (ubiquitin carboxy-terminal hydrolase)

BAX	NM_138761.3	BCL2-associated X protein
BCL2	NM_000657.2	B-cell CLL/lymphoma 2
BCL2A1	NM_004049.2	BCL2-related protein A1
BCL2L1	NM_138578.1	BCL2-like 1
BCOR	NM_001123383.1	BCL6 corepressor
BDNF	NM_170732.4	brain-derived neurotrophic factor
BID	NM_197966.1	BH3 interacting domain death agonist
BIRC3	NM_182962.1	baculoviral IAP repeat containing 3
BIRC7	NM_022161.2	baculoviral IAP repeat containing 7
BMP2	NM_001200.2	bone morphogenetic protein 2
BMP4	NM_001202.2	bone morphogenetic protein 4
BMP5	NM_021073.2	bone morphogenetic protein 5
BMP6	NM_001718.2	bone morphogenetic protein 6
BMP7	NM_001719.1	bone morphogenetic protein 7
BMP8A	NM_181809.3	bone morphogenetic protein 8a
BMPR1B	NM_001203.1	bone morphogenetic protein receptor, type IB
BNIP3	NM_004052.2	BCL2/adenovirus E1B 19kDa interacting protein 3
BRAF	NM_004333.3	v-raf murine sarcoma viral oncogene homolog B
BRCA1	NM_007305.2	breast cancer 1, early onset
BRCA2	NM_000059.3	breast cancer 2, early onset
BRIP1	NM_032043.1	BRCA1 interacting protein C-terminal helicase 1
C19orf40	NM_152266.3	chromosome 19 open reading frame 40
CACNA1C	NM_199460.2	calcium channel, voltage-dependent, L type, alpha 1C subunit
CACNA1D	NM_000720.2	calcium channel, voltage-dependent, L type, alpha 1D subunit
CACNA1E	NM_000721.2	calcium channel, voltage-dependent, R type, alpha 1E subunit
CACNA1G	NM_198397.1	calcium channel, voltage-dependent, T type, alpha 1G subunit
CACNA1H	NM_021098.2	calcium channel, voltage-dependent, T type, alpha 1H subunit
CACNA2D1	NM_000722.2	calcium channel, voltage-dependent, alpha 2/delta subunit 1
CACNA2D2	NM_001005505.1	calcium channel, voltage-dependent, alpha 2/delta subunit 2
CACNA2D3	NM_018398.2	calcium channel, voltage-dependent, alpha 2/delta subunit 3
CACNA2D4	NM_001005737.1	calcium channel, voltage-dependent, alpha 2/delta subunit 4
CACNB2	NM_000724.3	calcium channel, voltage-dependent, beta 2 subunit
CACNB3	NM_000725.2	calcium channel, voltage-dependent, beta 3 subunit
CACNB4	NM_001005747.2	calcium channel, voltage-dependent, beta 4 subunit
CACNG1	NM_000727.2	calcium channel, voltage-dependent, gamma subunit 1
CACNG4	NM_014405.2	calcium channel, voltage-dependent, gamma subunit 4
CACNG6	NM_145814.1	calcium channel, voltage-dependent, gamma subunit 6
CALML3	NM_005185.2	calmodulin-like 3
CALML5	NM_017422.4	calmodulin-like 5
CALML6	NM_138705.2	calmodulin-like 6
CAMK2B	NM_001220.3	calcium/calmodulin-dependent protein kinase II beta
CAPN2	NM_001748.4	calpain 2, (m/II) large subunit

CARD11	NM_032415.2	caspase recruitment domain family, member 11
CASP10	NM_032977.3	caspase 10, apoptosis-related cysteine peptidase
CASP12	NM_001191016.1	caspase 12 (gene/pseudogene)
CASP3	NM_032991.2	caspase 3, apoptosis-related cysteine peptidase
CASP7	NM_001227.3	caspase 7, apoptosis-related cysteine peptidase
CASP8	NM_001228.4	caspase 8, apoptosis-related cysteine peptidase
CASP9	NM_001229.2	caspase 9, apoptosis-related cysteine peptidase
CBL	NM_005188.2	Cbl proto-oncogene, E3 ubiquitin protein ligase
CBLC	NM_012116.3	Cbl proto-oncogene C, E3 ubiquitin protein ligase
CCNA1	NM_003914.3	cyclin A1
CCNA2	NM_001237.2	cyclin A2
CCNB1	NM_031966.2	cyclin B1
CCNB3	NM_033671.1	cyclin B3
CCND1	NM_053056.2	cyclin D1
CCND2	NM_001759.2	cyclin D2
CCND3	NM_001760.2	cyclin D3
CCNE1	NM_001238.1	cyclin E1
CCNE2	NM_057735.1	cyclin E2
CCNO	NM_021147.3	cyclin O
CCR7	NM_001838.2	chemokine (C-C motif) receptor 7
CD14	NM_000591.2	CD14 molecule
CD19	NM_001770.4	CD19 molecule
CD40	NM_001250.4	CD40 molecule, TNF receptor superfamily member 5
CDC14A	NM_033313.2	cell division cycle 14A
CDC14B	NM_003671.3	cell division cycle 14B
CDC25A	NM_001789.2	cell division cycle 25A
CDC25B	NM_021873.2	cell division cycle 25B
CDC25C	NM_001790.2	cell division cycle 25C
CDC6	NM_001254.3	cell division cycle 6
CDC7	NM_003503.2	cell division cycle 7
CDH1	NM_004360.2	cadherin 1, type 1, E-cadherin (epithelial)
CDK2	NM_001798.2	cyclin-dependent kinase 2
CDK4	NM_000075.2	cyclin-dependent kinase 4
CDK6	NM_001259.5	cyclin-dependent kinase 6
CDKN1A	NM_000389.2	cyclin-dependent kinase inhibitor 1A (p21, Cip1)
CDKN1B	NM_004064.2	cyclin-dependent kinase inhibitor 1B (p27, Kip1)
CDKN1C	NM_000076.2	cyclin-dependent kinase inhibitor 1C (p57, Kip2)
CDKN2A	NM_000077.3	cyclin-dependent kinase inhibitor 2A
CDKN2B	NM_004936.3	cyclin-dependent kinase inhibitor 2B (p15, inhibits CDK4)
CDKN2C	NM_001262.2	cyclin-dependent kinase inhibitor 2C (p18, inhibits CDK4)
CDKN2D	NM_001800.3	cyclin-dependent kinase inhibitor 2D (p19, inhibits CDK4)
CEBPA	NM_004364.2	CCAAT/enhancer binding protein (C/EBP), alpha

CEBPE	NM_001805.2	CCAAT/enhancer binding protein (C/EBP), epsilon
CHAD	NM_001267.2	Chondroadherin
CHEK1	NM_001114121.1	checkpoint kinase 1
CHEK2	NM_007194.3	checkpoint kinase 2
CHUK	NM_001278.3	conserved helix-loop-helix ubiquitous kinase
CIC	NM_015125.3	capicua transcriptional repressor
CLCF1	NM_013246.2	cardiotrophin-like cytokine factor 1
CNTFR	NM_147164.1	ciliary neurotrophic factor receptor
COL11A1	NM_001854.3	collagen, type XI, alpha 1
COL11A2	NM_001163771.1	collagen, type XI, alpha 2
COL1A1	NM_000088.3	collagen, type I, alpha 1
COL1A2	NM_000089.3	collagen, type I, alpha 2
COL24A1	NM_152890.5	collagen, type XXIV, alpha 1
COL27A1	NM_032888.2	collagen, type XXVII, alpha 1
COL2A1	NM_001844.4	collagen, type II, alpha 1
COL3A1	NM_000090.3	collagen, type III, alpha 1
COL4A3	NM_000091.3	collagen, type IV, alpha 3 (Goodpasture antigen)
COL4A4	NM_000092.4	collagen, type IV, alpha 4
COL4A5	NM_033381.1	collagen, type IV, alpha 5
COL4A6	NM_001847.2	collagen, type IV, alpha 6
COL5A1	NM_000093.3	collagen, type V, alpha 1
COL5A2	NM_000393.3	collagen, type V, alpha 2
COL6A6	NM_001102608.1	collagen, type VI, alpha 6
COMP	NM_000095.2	cartilage oligomeric matrix protein
CREB3L1	NM_052854.1	cAMP responsive element binding protein 3-like 1
CREB3L3	NM_001271995.1	cAMP responsive element binding protein 3-like 3
CREB3L4	NM_130898.2	cAMP responsive element binding protein 3-like 4
CREB5	NM_182898.2	cAMP responsive element binding protein 5
CREBBP	NM_004380.2	CREB binding protein
CRLF2	NM_001012288.1	cytokine receptor-like factor 2
CSF1R	NM_005211.2	colony stimulating factor 1 receptor
CSF2	NM_000758.2	colony stimulating factor 2 (granulocyte-macrophage)
CSF3	NM_000759.3	colony stimulating factor 3 (granulocyte)
CSF3R	NM_156038.2	colony stimulating factor 3 receptor (granulocyte)
CTNNB1	NM_001904.3	catenin (cadherin-associated protein), beta 1, 88kDa
CUL1	NM_003592.2	cullin 1
CXXC4	NM_025212.1	CXXC finger protein 4
CYLD	NM_015247.1	cylindromatosis (turban tumor syndrome)
DAXX	NM_001350.3	death-domain associated protein
DDB2	NM_000107.1	damage-specific DNA binding protein 2, 48kDa
DDIT3	NM_004083.4	DNA-damage-inducible transcript 3
DDIT4	NM_019058.2	DNA-damage-inducible transcript 4

DKK1	NM_012242.2	dickkopf WNT signaling pathway inhibitor 1
DKK2	NM_014421.2	dickkopf WNT signaling pathway inhibitor 2
DKK4	NM_014420.2	dickkopf WNT signaling pathway inhibitor 4
DLL1	NM_005618.3	delta-like 1 (Drosophila)
DLL3	NM_203486.2	delta-like 3 (Drosophila)
DLL4	NM_019074.2	delta-like 4 (Drosophila)
DNMT1	NM_001379.2	DNA (cytosine-5-)-methyltransferase 1
DNMT3A	NM_022552.3	DNA (cytosine-5-)-methyltransferase 3 alpha
DTX1	NM_004416.2	deltex 1, E3 ubiquitin ligase
DTX3	NM_178502.2	deltex 3, E3 ubiquitin ligase
DTX4	NM_015177.1	deltex 4, E3 ubiquitin ligase
DUSP10	NM_144728.2	dual specificity phosphatase 10
DUSP2	NM_004418.3	dual specificity phosphatase 2
DUSP4	NM_057158.2	dual specificity phosphatase 4
DUSP5	NM_004419.3	dual specificity phosphatase 5
DUSP6	NM_001946.2	dual specificity phosphatase 6
DUSP8	NM_004420.2	dual specificity phosphatase 8
E2F1	NM_005225.1	E2F transcription factor 1
E2F5	NM_001951.3	E2F transcription factor 5, p130-binding
EFNA1	NM_004428.2	ephrin-A1
EFNA2	NM_001405.3	ephrin-A2
EFNA3	NM_004952.4	ephrin-A3
EFNA5	NM_001962.2	ephrin-A5
EGF	NM_001963.3	epidermal growth factor
EGFR	NM_201282.1	epidermal growth factor receptor
EIF4EBP1	NM_004095.3	eukaryotic translation initiation factor 4E binding protein 1
ENDOG	NM_004435.2	endonuclease G
EP300	NM_001429.2	E1A binding protein p300
EPHA2	NM_004431.2	EPH receptor A2
EPO	NM_000799.2	Erythropoietin
EPOR	NM_000121.2	erythropoietin receptor
ERBB2	NM_004448.2	v-erb-b2 avian erythroblastic leukemia viral oncogene homolog 2
ERCC2	NM_000400.2	excision repair cross-complementing rodent repair deficiency, complementation group 2
ERCC6	NM_000124.2	excision repair cross-complementing rodent repair deficiency, complementation group 6
ETS2	NM_005239.4	v-ets avian erythroblastosis virus E26 oncogene homolog 2
ETV1	NM_004956.4	ets variant 1
ETV4	NM_001079675.1	ets variant 4
ETV7	NM_016135.2	ets variant 7
EYA1	NM_172059.2	eyes absent homolog 1 (Drosophila)
EZH2	NM_004456.3	enhancer of zeste homolog 2 (Drosophila)
FANCA	NM_000135.2	Fanconi anemia, complementation group A

FANCB	NM_152633.2	Fanconi anemia, complementation group B
FANCC	NM_000136.2	Fanconi anemia, complementation group C
FANCE	NM_021922.2	Fanconi anemia, complementation group E
FANCF	NM_022725.2	Fanconi anemia, complementation group F
FANCG	NM_004629.1	Fanconi anemia, complementation group G
FANCL	NM_001114636.1	Fanconi anemia, complementation group L
FAS	NM_152876.1	Fas cell surface death receptor
FASLG	NM_000639.1	Fas ligand (TNF superfamily, member 6)
FBXW7	NM_018315.4	F-box and WD repeat domain containing 7, E3 ubiquitin protein ligase
FEN1	NM_004111.4	flap structure-specific endonuclease 1
FGF1	NM_033137.1	fibroblast growth factor 1 (acidic)
FGF10	NM_004465.1	fibroblast growth factor 10
FGF11	NM_004112.2	fibroblast growth factor 11
FGF12	NM_004113.4	fibroblast growth factor 12
FGF13	NM_033642.1	fibroblast growth factor 13
FGF14	NM_004115.3	fibroblast growth factor 14
FGF16	NM_003868.1	fibroblast growth factor 16
FGF17	NM_003867.2	fibroblast growth factor 17
FGF18	NM_003862.1	fibroblast growth factor 18
FGF19	NM_005117.2	fibroblast growth factor 19
FGF2	NM_002006.4	fibroblast growth factor 2 (basic)
FGF20	NM_019851.1	fibroblast growth factor 20
FGF21	NM_019113.2	fibroblast growth factor 21
FGF22	NM_020637.1	fibroblast growth factor 22
FGF23	NM_020638.2	fibroblast growth factor 23
FGF3	NM_005247.2	fibroblast growth factor 3
FGF4	NM_002007.2	fibroblast growth factor 4
FGF5	NM_004464.3	fibroblast growth factor 5
FGF6	NM_020996.1	fibroblast growth factor 6
FGF7	NM_002009.3	fibroblast growth factor 7
FGF8	NM_033163.3	fibroblast growth factor 8 (androgen-induced)
FGF9	NM_002010.2	fibroblast growth factor 9
FGFR1	NM_015850.2	fibroblast growth factor receptor 1
FGFR2	NM_000141.4	fibroblast growth factor receptor 2
FGFR3	NM_022965.2	fibroblast growth factor receptor 3
FGFR4	NM_002011.3	fibroblast growth factor receptor 4
FIGF	NM_004469.2	c-fos induced growth factor (vascular endothelial growth factor D)
FLNA	NM_001456.3	filamin A, alpha
FLNC	NM_001127487.1	filamin C, gamma
FLT1	NM_002019.4	fms-related tyrosine kinase 1
FLT3	NM_004119.1	fms-related tyrosine kinase 3

FN1	NM_212482.1	fibronectin 1
FOS	NM_005252.2	FBJ murine osteosarcoma viral oncogene homolog
FOSL1	NM_005438.2	FOS-like antigen 1
FOXL2	NM_023067.2	forkhead box L2
FOXO4	NM_005938.2	forkhead box O4
FST	NM_006350.2	Follistatin
FUBP1	NM_003902.3	far upstream element (FUSE) binding protein 1
FUT8	NM_004480.4	fucosyltransferase 8 (alpha (1,6) fucosyltransferase)
FZD10	NM_007197.2	frizzled family receptor 10
FZD2	NM_001466.2	frizzled family receptor 2
FZD3	NM_017412.2	frizzled family receptor 3
FZD7	NM_003507.1	frizzled family receptor 7
FZD8	NM_031866.1	frizzled family receptor 8
FZD9	NM_003508.2	frizzled family receptor 9
GADD45A	NM_001924.2	growth arrest and DNA-damage-inducible, alpha
GADD45B	NM_015675.2	growth arrest and DNA-damage-inducible, beta
GADD45G	NM_006705.3	growth arrest and DNA-damage-inducible, gamma
GAS1	NM_002048.2	growth arrest-specific 1
GATA1	NM_002049.2	GATA binding protein 1 (globin transcription factor 1)
GATA2	NM_032638.3	GATA binding protein 2
GATA3	NM_001002295.1	GATA binding protein 3
GDF6	NM_001001557.2	growth differentiation factor 6
GHR	NM_000163.2	growth hormone receptor
GLI1	NM_005269.1	GLI family zinc finger 1
GLI3	NM_000168.5	GLI family zinc finger 3
GNA11	NM_002067.1	guanine nucleotide binding protein (G protein), alpha 11 (Gq class)
GNAQ	NM_002072.2	guanine nucleotide binding protein (G protein), q polypeptide
GNAS	NM_080425.1	GNAS complex locus
GNG12	NM_018841.3	guanine nucleotide binding protein (G protein), gamma 12
GNG4	NM_004485.2	guanine nucleotide binding protein (G protein), gamma 4
GNG7	NM_052847.1	guanine nucleotide binding protein (G protein), gamma 7
GNGT1	NM_021955.3	guanine nucleotide binding protein (G protein), gamma transducing activity polypeptide 1
GPC4	NM_001448.2	glypican 4
GRB2	NM_002086.4	growth factor receptor-bound protein 2
GRIA3	NM_000828.4	glutamate receptor, ionotropic, AMPA 3
GRIN1	NM_000832.5	glutamate receptor, ionotropic, N-methyl D-aspartate 1
GRIN2A	NM_000833.3	glutamate receptor, ionotropic, N-methyl D-aspartate 2A
GRIN2B	NM_000834.3	glutamate receptor, ionotropic, N-methyl D-aspartate 2B
GSK3B	NM_002093.2	glycogen synthase kinase 3 beta
GTF2H3	NM_001516.3	general transcription factor IIH, polypeptide 3, 34kDa

GZMB	NM_004131.3	granzyme B (granzyme 2, cytotoxic T-lymphocyte-associated serine esterase 1)
H2AFX	NM_002105.2	H2A histone family, member X
H3F3A	NM_002107.3	H3 histone, family 3A
H3F3C	NM_001013699.2	H3 histone, family 3C
HDAC1	NM_004964.2	histone deacetylase 1
HDAC10	NM_032019.5	histone deacetylase 10
HDAC11	NM_024827.3	histone deacetylase 11
HDAC2	NM_001527.1	histone deacetylase 2
HDAC4	NM_006037.3	histone deacetylase 4
HDAC5	NM_005474.4	histone deacetylase 5
HDAC6	NM_006044.2	histone deacetylase 6
HELLS	NM_018063.3	helicase, lymphoid-specific
HES1	NM_005524.2	hes family bHLH transcription factor 1
HES5	NM_001010926.3	hes family bHLH transcription factor 5
HGF	NM_000601.4	hepatocyte growth factor (hepapoietin A; scatter factor)
HHEX	NM_002729.4	hematopoietically expressed homeobox
HHIP	NM_022475.1	hedgehog interacting protein
HIST1H3B	NM_003537.3	histone cluster 1, H3b
HIST1H3G	NM_003534.2	histone cluster 1, H3g
HIST1H3H	NM_003536.2	histone cluster 1, H3h
HMGA1	NM_145904.1	high mobility group AT-hook 1
HMGA2	NM_003484.1	high mobility group AT-hook 2
HNF1A	NM_000545.4	HNF1 homeobox A
HOXA10	NM_018951.3	homeobox A10
HOXA11	NM_005523.5	homeobox A11
HOXA9	NM_152739.3	homeobox A9
HPGD	NM_001145816.2	hydroxyprostaglandin dehydrogenase 15-(NAD)
HRAS	NM_005343.2	Harvey rat sarcoma viral oncogene homolog
HSP90B1	NM_003299.1	heat shock protein 90kDa beta (Grp94), member 1
HSPA1A	NM_005345.5	heat shock 70kDa protein 1A
HSPA2	NM_021979.3	heat shock 70kDa protein 2
HSPA6	NM_002155.3	heat shock 70kDa protein 6 (HSP70B')
HSPB1	NM_001540.3	heat shock 27kDa protein 1
IBSP	NM_004967.3	integrin-binding sialoprotein
ID1	NM_002165.2	inhibitor of DNA binding 1, dominant negative helix-loop-helix protein
ID2	NM_002166.4	inhibitor of DNA binding 2, dominant negative helix-loop-helix protein
ID4	NM_001546.2	inhibitor of DNA binding 4, dominant negative helix-loop-helix protein
IDH1	NM_005896.2	isocitrate dehydrogenase 1 (NADP+), soluble
IDH2	NM_002168.2	isocitrate dehydrogenase 2 (NADP+), mitochondrial

IFNA17	NM_021268.2	interferon, alpha 17
IFNA2	NM_000605.3	interferon, alpha 2
IFNA7	NM_021057.2	interferon, alpha 7
IFNG	NM_000619.2	interferon, gamma
IGF1	NM_000618.3	insulin-like growth factor 1 (somatomedin C)
IGF1R	NM_000875.2	insulin-like growth factor 1 receptor
IGFBP3	NM_000598.4	insulin-like growth factor binding protein 3
IKBKB	NM_001556.1	inhibitor of kappa light polypeptide gene enhancer in B-cells, kinase beta
IKBKG	NM_003639.2	inhibitor of kappa light polypeptide gene enhancer in B-cells, kinase gamma
IL10	NM_000572.2	interleukin 10
IL11	NM_000641.2	interleukin 11
IL11RA	NM_147162.1	interleukin 11 receptor, alpha
IL12A	NM_000882.2	interleukin 12A (natural killer cell stimulatory factor 1, cytotoxic lymphocyte maturation factor 1, p35)
IL12B	NM_002187.2	interleukin 12B (natural killer cell stimulatory factor 2, cytotoxic lymphocyte maturation factor 2, p40)
IL12RB2	NM_001559.2	interleukin 12 receptor, beta 2
IL13	NM_002188.2	interleukin 13
IL13RA2	NM_000640.2	interleukin 13 receptor, alpha 2
IL15	NM_172174.1	interleukin 15
IL19	NM_013371.3	interleukin 19
IL1A	NM_000575.3	interleukin 1, alpha
IL1B	NM_000576.2	interleukin 1, beta
IL1R1	NM_000877.2	interleukin 1 receptor, type I
IL1R2	NM_173343.1	interleukin 1 receptor, type II
IL1RAP	NM_002182.2	interleukin 1 receptor accessory protein
IL20RA	NM_014432.2	interleukin 20 receptor, alpha
IL20RB	NM_144717.2	interleukin 20 receptor beta
IL22RA1	NM_021258.2	interleukin 22 receptor, alpha 1
IL22RA2	NM_181309.1	interleukin 22 receptor, alpha 2
IL23A	NM_016584.2	interleukin 23, alpha subunit p19
IL23R	NM_144701.2	interleukin 23 receptor
IL24	NM_181339.1	interleukin 24
IL2RA	NM_000417.1	interleukin 2 receptor, alpha
IL2RB	NM_000878.2	interleukin 2 receptor, beta
IL3	NM_000588.3	interleukin 3 (colony-stimulating factor, multiple)
IL3RA	NM_002183.2	interleukin 3 receptor, alpha (low affinity)
IL5RA	NM_000564.3	interleukin 5 receptor, alpha
IL6	NM_000600.1	interleukin 6 (interferon, beta 2)
IL6R	NM_000565.2	interleukin 6 receptor
IL7	NM_000880.2	interleukin 7

IL7R	NM_002185.2	interleukin 7 receptor
IL8	NM_000584.2	interleukin 8
INHBA	NM_002192.2	inhibin, beta A
INHBB	NM_002193.2	inhibin, beta B
IRAK2	NM_001570.3	interleukin-1 receptor-associated kinase 2
IRAK3	NM_007199.1	interleukin-1 receptor-associated kinase 3
IRS1	NM_005544.2	insulin receptor substrate 1
ITGA2	NM_002203.2	integrin, alpha 2 (CD49B, alpha 2 subunit of VLA-2 receptor)
ITGA3	NM_005501.2	integrin, alpha 3 (antigen CD49C, alpha 3 subunit of VLA-3 receptor)
ITGA6	NM_000210.1	integrin, alpha 6
ITGA7	NM_002206.1	integrin, alpha 7
ITGA8	NM_003638.1	integrin, alpha 8
ITGA9	NM_002207.2	integrin, alpha 9
ITGB3	NM_000212.2	integrin, beta 3 (platelet glycoprotein IIIa, antigen CD61)
ITGB4	NM_001005731.1	integrin, beta 4
ITGB6	NM_001282353.1	integrin, beta 6
ITGB7	NM_000889.1	integrin, beta 7
ITGB8	NM_002214.2	integrin, beta 8
JAG1	NM_000214.2	jagged 1
JAG2	NM_145159.1	jagged 2
JAK1	NM_002227.1	Janus kinase 1
JAK2	NM_004972.2	Janus kinase 2
JAK3	NM_000215.2	Janus kinase 3
JUN	NM_002228.3	jun proto-oncogene
KAT2B	NM_003884.3	K(lysine) acetyltransferase 2B
KDM5C	NM_004187.2	lysine (K)-specific demethylase 5C
KDM6A	NM_021140.2	lysine (K)-specific demethylase 6A
KIT	NM_000222.1	v-kit Hardy-Zuckerman 4 feline sarcoma viral oncogene homolog
KITLG	NM_003994.4	KIT ligand
KLF4	NM_004235.4	Kruppel-like factor 4 (gut)
KMT2C	NM_170606.2	lysine (K)-specific methyltransferase 2C
KMT2D	NM_003482.3	lysine (K)-specific methyltransferase 2D
KRAS	NM_004985.3	Kirsten rat sarcoma viral oncogene homolog
LAMA1	NM_005559.2	laminin, alpha 1
LAMA3	NM_000227.3	laminin, alpha 3
LAMA5	NM_005560.3	laminin, alpha 5
LAMB3	NM_000228.2	laminin, beta 3
LAMB4	NM_007356.2	laminin, beta 4
LAMC2	NM_005562.2	laminin, gamma 2
LAMC3	NM_006059.3	laminin, gamma 3
LAT	NM_001014987.1	linker for activation of T cells

LEF1	NM_016269.3	lymphoid enhancer-binding factor 1
LEFTY1	NM_020997.2	left-right determination factor 1
LEFTY2	NM_003240.2	left-right determination factor 2
LEP	NM_000230.2	Leptin
LEPR	NM_001003679.1	leptin receptor
LFNG	NM_001040168.1	LFNG O-fucosylpeptide 3-beta-N-acetylglucosaminyltransferase
LIF	NM_002309.3	leukemia inhibitory factor
LIFR	NM_002310.3	leukemia inhibitory factor receptor alpha
LIG4	NM_002312.3	ligase IV, DNA, ATP-dependent
LRP2	NM_004525.2	low density lipoprotein receptor-related protein 2
LTBP1	NM_000627.3	latent transforming growth factor beta binding protein 1
MAD2L2	NM_001127325.1	MAD2 mitotic arrest deficient-like 2 (yeast)
MAML2	NM_032427.1	mastermind-like 2 (Drosophila)
MAP2K1	NM_002755.2	mitogen-activated protein kinase kinase 1
MAP2K2	NM_030662.2	mitogen-activated protein kinase kinase 2
MAP2K4	NM_003010.2	mitogen-activated protein kinase kinase 4
MAP2K6	NM_002758.3	mitogen-activated protein kinase kinase 6
MAP3K1	NM_005921.1	mitogen-activated protein kinase kinase kinase 1, E3 ubiquitin protein ligase
MAP3K12	NM_006301.2	mitogen-activated protein kinase kinase kinase 12
MAP3K13	NM_004721.3	mitogen-activated protein kinase kinase kinase 13
MAP3K14	NM_003954.1	mitogen-activated protein kinase kinase kinase 14
MAP3K5	NM_005923.3	mitogen-activated protein kinase kinase kinase 5
MAP3K8	NM_005204.2	mitogen-activated protein kinase kinase kinase 8
MAPK1	NM_138957.2	mitogen-activated protein kinase 1
MAPK10	NM_002753.2	mitogen-activated protein kinase 10
MAPK12	NM_002969.3	mitogen-activated protein kinase 12
MAPK3	NM_001040056.1	mitogen-activated protein kinase 3
MAPK8	NM_002750.2	mitogen-activated protein kinase 8
MAPK8IP1	NM_005456.2	mitogen-activated protein kinase 8 interacting protein 1
MAPK8IP2	NM_012324.2	mitogen-activated protein kinase 8 interacting protein 2
MAPK9	NM_139068.2	mitogen-activated protein kinase 9
MAPT	NM_016834.3	microtubule-associated protein tau
MCM2	NM_004526.2	minichromosome maintenance complex component 2
MCM4	NM_182746.1	minichromosome maintenance complex component 4
MCM5	NM_006739.3	minichromosome maintenance complex component 5
MCM7	NM_182776.1	minichromosome maintenance complex component 7
MDC1	NM_014641.2	mediator of DNA-damage checkpoint 1
MDM2	NM_006878.2	MDM2 oncogene, E3 ubiquitin protein ligase
MECOM	NM_005241.2	MDS1 and EVI1 complex locus
MED12	NM_005120.2	mediator complex subunit 12
MEN1	NM_130802.2	multiple endocrine neoplasia I

MET	NM_000245.2	met proto-oncogene
MFNG	NM_002405.2	MFNG O-fucosylpeptide 3-beta-N-acetylglucosaminyltransferase
MGMT	NM_002412.3	O-6-methylguanine-DNA methyltransferase
MLF1	NM_022443.3	myeloid leukemia factor 1
MLH1	NM_000249.2	mutL homolog 1
MLLT3	NM_004529.2	myeloid/lymphoid or mixed-lineage leukemia (trithorax homolog, Drosophila); translocated to, 3
MLLT4	NM_005936.2	myeloid/lymphoid or mixed-lineage leukemia (trithorax homolog, Drosophila); translocated to, 4
MMP3	NM_002422.3	matrix metallopeptidase 3 (stromelysin 1, progelatinase)
MMP7	NM_002423.3	matrix metallopeptidase 7 (matrilysin, uterine)
MMP9	NM_004994.2	matrix metallopeptidase 9 (gelatinase B, 92kDa gelatinase, 92kDa type IV collagenase)
MNAT1	NM_002431.2	MNAT CDK-activating kinase assembly factor 1
MPL	NM_005373.2	myeloproliferative leukemia virus oncogene
MPO	NM_000250.1	Myeloperoxidase
MSH2	NM_000251.1	mutS homolog 2
MSH6	NM_000179.1	mutS homolog 6
MTOR	NM_004958.2	mechanistic target of rapamycin (serine/threonine kinase)
MUTYH	NM_012222.2	mutY homolog
MYB	NM_005375.2	v-myb avian myeloblastosis viral oncogene homolog
MYC	NM_002467.3	v-myc avian myelocytomatosis viral oncogene homolog
MYCN	NM_005378.4	v-myc avian myelocytomatosis viral oncogene neuroblastoma derived homolog
MYD88	NM_002468.3	myeloid differentiation primary response 88
NASP	NM_172164.1	nuclear autoantigenic sperm protein (histone-binding)
NBN	NM_001024688.1	Nibrin
NCOR1	NM_006311.3	nuclear receptor corepressor 1
NF1	NM_000267.2	neurofibromin 1
NF2	NM_181828.2	neurofibromin 2 (merlin)
NFATC1	NM_172389.1	nuclear factor of activated T-cells, cytoplasmic, calcineurin-dependent 1
NFE2L2	NM_006164.3	nuclear factor, erythroid 2-like 2
NFKB1	NM_003998.2	nuclear factor of kappa light polypeptide gene enhancer in B-cells 1
NFKBIA	NM_020529.1	nuclear factor of kappa light polypeptide gene enhancer in B-cells inhibitor, alpha
NFKBIZ	NM_001005474.1	nuclear factor of kappa light polypeptide gene enhancer in B-cells inhibitor, zeta
NGF	NM_002506.2	nerve growth factor (beta polypeptide)
NGFR	NM_002507.1	nerve growth factor receptor
NKD1	NM_033119.3	naked cuticle homolog 1 (Drosophila)
NODAL	NM_018055.3	nodal growth differentiation factor
NOG	NM_005450.4	Noggin

NOS3	NM_000603.4	nitric oxide synthase 3 (endothelial cell)
NOTCH1	NM_017617.3	notch 1
NOTCH2	NM_024408.3	notch 2
NOTCH3	NM_000435.2	notch 3
NPM1	NM_002520.5	nucleophosmin (nucleolar phosphoprotein B23, numatrin)
NPM2	NM_182795.1	nucleophosmin/nucleoplasmin 2
NR4A1	NM_173157.1	nuclear receptor subfamily 4, group A, member 1
NR4A3	NM_173198.1	nuclear receptor subfamily 4, group A, member 3
NRAS	NM_002524.3	neuroblastoma RAS viral (v-ras) oncogene homolog
NSD1	NM_022455.4	nuclear receptor binding SET domain protein 1
NTF3	NM_002527.4	neurotrophin 3
NTHL1	NM_002528.5	nth endonuclease III-like 1 (E. coli)
NTRK1	NM_001012331.1	neurotrophic tyrosine kinase, receptor, type 1
NTRK2	NM_001007097.1	neurotrophic tyrosine kinase, receptor, type 2
NUMBL	NM_004756.3	numb homolog (Drosophila)-like
NUPR1	NM_001042483.1	nuclear protein, transcriptional regulator, 1
OSM	NM_020530.3	oncostatin M
PAK3	NM_002578.2	p21 protein (Cdc42/Rac)-activated kinase 3
PAK7	NM_177990.1	p21 protein (Cdc42/Rac)-activated kinase 7
PAX3	NM_013942.3	paired box 3
PAX5	NM_016734.1	paired box 5
PAX8	NM_013953.3	paired box 8
PBRM1	NM_181042.3	polybromo 1
PBX1	NM_002585.2	pre-B-cell leukemia homeobox 1
PBX3	NM_006195.5	pre-B-cell leukemia homeobox 3
PCK1	NM_002591.2	phosphoenolpyruvate carboxykinase 1 (soluble)
PCNA	NM_002592.2	proliferating cell nuclear antigen
PDGFA	NM_002607.5	platelet-derived growth factor alpha polypeptide
PDGFB	NM_033016.2	platelet-derived growth factor beta polypeptide
PDGFC	NM_016205.1	platelet derived growth factor C
PDGFD	NM_025208.4	platelet derived growth factor D
PDGFRA	NM_006206.3	platelet-derived growth factor receptor, alpha polypeptide
PDGFRB	NM_002609.3	platelet-derived growth factor receptor, beta polypeptide
PGF	NM_002632.5	placental growth factor
PHF6	NM_032335.3	PHD finger protein 6
PIK3CA	NM_006218.2	phosphatidylinositol-4,5-bisphosphate 3-kinase, catalytic subunit alpha
PIK3CB	NM_006219.1	phosphatidylinositol-4,5-bisphosphate 3-kinase, catalytic subunit beta
PIK3CD	NM_005026.3	phosphatidylinositol-4,5-bisphosphate 3-kinase, catalytic subunit delta
PIK3CG	NM_002649.2	phosphatidylinositol-4,5-bisphosphate 3-kinase, catalytic subunit gamma

PIK3R1	NM_181504.2	phosphoinositide-3-kinase, regulatory subunit 1 (alpha)
PIK3R2	NM_005027.2	phosphoinositide-3-kinase, regulatory subunit 2 (beta)
PIK3R3	NM_003629.3	phosphoinositide-3-kinase, regulatory subunit 3 (gamma)
PIK3R5	NM_001142633.1	phosphoinositide-3-kinase, regulatory subunit 5
PIM1	NM_002648.2	pim-1 oncogene
PITX2	NM_000325.5	paired-like homeodomain 2
PKMYT1	NM_004203.3	protein kinase, membrane associated tyrosine/threonine 1
PLA1A	NM_015900.2	phospholipase A1 member A
PLA2G10	NM_003561.1	phospholipase A2, group X
PLA2G2A	NM_000300.2	phospholipase A2, group IIA (platelets, synovial fluid)
PLA2G3	NM_015715.3	phospholipase A2, group III
PLA2G4A	NM_024420.2	phospholipase A2, group IVA (cytosolic, calcium-dependent)
PLA2G4C	NM_003706.2	phospholipase A2, group IVC (cytosolic, calcium-independent)
PLA2G4E	NM_001206670.1	phospholipase A2, group IVE
PLA2G4F	NM_213600.2	phospholipase A2, group IVF
PLA2G5	NM_000929.2	phospholipase A2, group V
PLAT	NM_000931.2	plasminogen activator, tissue
PLAU	NM_002658.2	plasminogen activator, urokinase
PLCB1	NM_182734.1	phospholipase C, beta 1 (phosphoinositide-specific)
PLCB4	NM_000933.3	phospholipase C, beta 4
PLCE1	NM_001165979.1	phospholipase C, epsilon 1
PLCG2	NM_002661.2	phospholipase C, gamma 2 (phosphatidylinositol-specific)
PLD1	NM_002662.3	phospholipase D1, phosphatidylcholine-specific
PML	NM_002675.3	promyelocytic leukemia
POLB	NM_002690.1	polymerase (DNA directed), beta
POLD1	NM_002691.2	polymerase (DNA directed), delta 1, catalytic subunit
POLD4	NM_021173.2	polymerase (DNA-directed), delta 4, accessory subunit
POLE2	NM_002692.2	polymerase (DNA directed), epsilon 2, accessory subunit
POLR2D	NM_004805.3	polymerase (RNA) II (DNA directed) polypeptide D
POLR2H	NM_001278698.1	polymerase (RNA) II (DNA directed) polypeptide H
POLR2J	NM_006234.4	polymerase (RNA) II (DNA directed) polypeptide J, 13.3kDa
PPARG	NM_015869.3	peroxisome proliferator-activated receptor gamma
PPARGC1A	NM_013261.3	peroxisome proliferator-activated receptor gamma, coactivator 1 alpha
PPP2CB	NM_001009552.1	protein phosphatase 2, catalytic subunit, beta isozyme
PPP2R1A	NM_014225.3	protein phosphatase 2, regulatory subunit A, alpha
PPP2R2B	NM_181676.2	protein phosphatase 2, regulatory subunit B, beta
PPP2R2C	NM_181876.2	protein phosphatase 2, regulatory subunit B, gamma
PPP3CA	NM_000944.4	protein phosphatase 3, catalytic subunit, alpha isozyme
PPP3CB	NM_001142354.1	protein phosphatase 3, catalytic subunit, beta isozyme
PPP3CC	NM_005605.3	protein phosphatase 3, catalytic subunit, gamma isozyme
PPP3R1	NM_000945.3	protein phosphatase 3, regulatory subunit B, alpha

PPP3R2	NM_147180.2	protein phosphatase 3, regulatory subunit B, beta
PRDM1	NM_182907.1	PR domain containing 1, with ZNF domain
PRKAA2	NM_006252.2	protein kinase, AMP-activated, alpha 2 catalytic subunit
PRKACA	NM_002730.3	protein kinase, cAMP-dependent, catalytic, alpha
PRKACB	NM_182948.2	protein kinase, cAMP-dependent, catalytic, beta
PRKACG	NM_002732.2	protein kinase, cAMP-dependent, catalytic, gamma
PRKAR1B	NM_001164759.1	protein kinase, cAMP-dependent, regulatory, type I, beta
PRKAR2A	NM_004157.2	protein kinase, cAMP-dependent, regulatory, type II, alpha
PRKAR2B	NM_002736.2	protein kinase, cAMP-dependent, regulatory, type II, beta
PRKCA	NM_002737.2	protein kinase C, alpha
PRKCB	NM_212535.1	protein kinase C, beta
PRKCG	NM_002739.3	protein kinase C, gamma
PRKDC	NM_006904.6	protein kinase, DNA-activated, catalytic polypeptide
PRKX	NM_005044.1	protein kinase, X-linked
PRL	NM_000948.3	Prolactin
PRLR	NM_001204318.1	prolactin receptor
PRMT8	NM_019854.3	protein arginine methyltransferase 8
PROM1	NM_006017.1	prominin 1
PTCH1	NM_000264.3	patched 1
PTCRA	NM_138296.2	pre T-cell antigen receptor alpha
PTEN	NM_000314.3	phosphatase and tensin homolog
PTPN11	NM_002834.3	protein tyrosine phosphatase, non-receptor type 11
PTPN5	NM_001039970.1	protein tyrosine phosphatase, non-receptor type 5 (striatum-enriched)
PTPRR	NM_001207015.1	protein tyrosine phosphatase, receptor type, R
PTTG2	NM_006607.2	pituitary tumor-transforming 2
RAC1	NM_198829.1	ras-related C3 botulinum toxin substrate 1 (rho family, small GTP binding protein Rac1)
RAC2	NM_002872.3	ras-related C3 botulinum toxin substrate 2 (rho family, small GTP binding protein Rac2)
RAC3	NM_005052.2	ras-related C3 botulinum toxin substrate 3 (rho family, small GTP binding protein Rac3)
RAD21	NM_006265.2	RAD21 homolog (S. pombe)
RAD50	NM_005732.2	RAD50 homolog (S. cerevisiae)
RAD51	NM_133487.2	RAD51 recombinase
RAD52	NM_134424.2	RAD52 homolog (S. cerevisiae)
RAF1	NM_002880.2	v-raf-1 murine leukemia viral oncogene homolog 1
RASA4	NM_001079877.2	RAS p21 protein activator 4
RASAL1	NM_004658.1	RAS protein activator like 1 (GAP1 like)
RASGRF1	NM_153815.2	Ras protein-specific guanine nucleotide-releasing factor 1
RASGRF2	NM_006909.1	Ras protein-specific guanine nucleotide-releasing factor 2
RASGRP1	NM_005739.3	RAS guanyl releasing protein 1 (calcium and DAG-regulated)
RASGRP2	NM_001098670.1	RAS guanyl releasing protein 2 (calcium and DAG-regulated)

RB1	NM_000321.1	retinoblastoma 1
RBX1	NM_014248.2	ring-box 1, E3 ubiquitin protein ligase
RELA	NM_021975.2	v-rel avian reticuloendotheliosis viral oncogene homolog A
RELN	NM_005045.2	Reelin
RET	NM_020630.4	ret proto-oncogene
RFC3	NM_002915.3	replication factor C (activator 1) 3, 38kDa
RFC4	NM_181573.2	replication factor C (activator 1) 4, 37kDa
RHOA	NM_001664.2	ras homolog family member A
RIN1	NM_004292.2	Ras and Rab interactor 1
RNF43	NM_017763.4	ring finger protein 43
RPA3	NM_002947.3	replication protein A3, 14kDa
RPS27A	NM_002954.5	ribosomal protein S27a
RPS6KA5	NM_004755.2	ribosomal protein S6 kinase, 90kDa, polypeptide 5
RPS6KA6	NM_014496.1	ribosomal protein S6 kinase, 90kDa, polypeptide 6
RRAS2	NM_001102669.2	related RAS viral (r-ras) oncogene homolog 2
RUNX1	NM_001754.4	runt-related transcription factor 1
RUNX1T1	NM_004349.2	runt-related transcription factor 1; translocated to, 1 (cyclin D-related)
RXRG	NM_006917.3	retinoid X receptor, gamma
SETBP1	NM_015559.2	SET binding protein 1
SETD2	NM_014159.6	SET domain containing 2
SF3B1	NM_001005526.1	splicing factor 3b, subunit 1, 155kDa
SFN	NM_006142.3	Stratifin
SFRP1	NM_003012.3	secreted frizzled-related protein 1
SFRP2	NM_003013.2	secreted frizzled-related protein 2
SFRP4	NM_003014.2	secreted frizzled-related protein 4
SGK2	NM_170693.1	serum/glucocorticoid regulated kinase 2
SHC1	NM_183001.4	SHC (Src homology 2 domain containing) transforming protein 1
SHC2	NM_012435.2	SHC (Src homology 2 domain containing) transforming protein 2
SHC3	NM_016848.5	SHC (Src homology 2 domain containing) transforming protein 3
SHC4	NM_203349.2	SHC (Src homology 2 domain containing) family, member 4
SIN3A	NM_015477.1	SIN3 transcription regulator family member A
SIRT4	NM_012240.1	sirtuin 4
SIX1	NM_005982.3	SIX homeobox 1
SKP1	NM_170679.2	S-phase kinase-associated protein 1
SKP2	NM_005983.2	S-phase kinase-associated protein 2, E3 ubiquitin protein ligase
SMAD2	NM_001003652.1	SMAD family member 2
SMAD3	NM_005902.3	SMAD family member 3
SMAD4	NM_005359.3	SMAD family member 4
SMAD9	NM_005905.2	SMAD family member 9
SMARCA4	NM_003072.3	SWI/SNF related, matrix associated, actin dependent regulator of chromatin, subfamily a, member 4

SMARCB1	NM_003073.3	SWI/SNF related, matrix associated, actin dependent regulator of chromatin, subfamily b, member 1
SMC1A	NM_006306.2	structural maintenance of chromosomes 1A
SMC1B	NM_148674.3	structural maintenance of chromosomes 1B
SMC3	NM_005445.3	structural maintenance of chromosomes 3
SMO	NM_005631.3	smoothened, frizzled family receptor
SOCS1	NM_003745.1	suppressor of cytokine signaling 1
SOCS2	NM_003877.3	suppressor of cytokine signaling 2
SOCS3	NM_003955.3	suppressor of cytokine signaling 3
SOS1	NM_005633.2	son of sevenless homolog 1 (Drosophila)
SOS2	NM_006939.2	son of sevenless homolog 2 (Drosophila)
SOST	NM_025237.2	Sclerostin
SOX17	NM_022454.3	SRY (sex determining region Y)-box 17
SOX9	NM_000346.2	SRY (sex determining region Y)-box 9
SP1	NM_003109.1	Sp1 transcription factor
SPOP	NM_001007226.1	speckle-type POZ protein
SPP1	NM_000582.2	secreted phosphoprotein 1
SPRY1	NM_005841.1	sprouty homolog 1, antagonist of FGF signaling (Drosophila)
SPRY2	NM_005842.2	sprouty homolog 2 (Drosophila)
SPRY4	NM_030964.3	sprouty homolog 4 (Drosophila)
SRSF2	NM_003016.3	serine/arginine-rich splicing factor 2
SSX1	NM_005635.2	synovial sarcoma, X breakpoint 1
STAG2	NM_001042749.1	stromal antigen 2
STAT1	NM_007315.2	signal transducer and activator of transcription 1, 91kDa
STAT3	NM_139276.2	signal transducer and activator of transcription 3 (acute-phase response factor)
STAT4	NM_003151.2	signal transducer and activator of transcription 4
STK11	NM_000455.4	serine/threonine kinase 11
STMN1	NM_203401.1	stathmin 1
SUV39H2	NM_024670.3	suppressor of variegation 3-9 homolog 2 (Drosophila)
SYK	NM_003177.3	spleen tyrosine kinase
TBL1XR1	NM_024665.4	transducin (beta)-like 1 X-linked receptor 1
TCF3	NM_003200.2	transcription factor 3
TCF7L1	NM_031283.1	transcription factor 7-like 1 (T-cell specific, HMG-box)
TCL1B	NM_004918.2	T-cell leukemia/lymphoma 1B
TET2	NM_001127208.2	tet methylcytosine dioxygenase 2
TFDP1	NM_007111.4	transcription factor Dp-1
TGFB1	NM_000660.3	transforming growth factor, beta 1
TGFB2	NM_003238.2	transforming growth factor, beta 2
TGFB3	NM_003239.2	transforming growth factor, beta 3
TGFBR2	NM_001024847.1	transforming growth factor, beta receptor II (70/80kDa)
THBS1	NM_003246.2	thrombospondin 1
THBS4	NM_003248.3	thrombospondin 4

THEM4	NM_053055.4	thioesterase superfamily member 4
TIAM1	NM_003253.2	T-cell lymphoma invasion and metastasis 1
TLR2	NM_003264.3	toll-like receptor 2
TLR4	NM_138554.2	toll-like receptor 4
TLX1	NM_005521.3	T-cell leukemia homeobox 1
TMPRSS2	NM_005656.2	transmembrane protease, serine 2
TNC	NM_002160.3	tenascin C
TNF	NM_000594.2	tumor necrosis factor
TNFAIP3	NM_006290.2	tumor necrosis factor, alpha-induced protein 3
TNFRSF10A	NM_003844.2	tumor necrosis factor receptor superfamily, member 10a
TNFRSF10B	NM_003842.3	tumor necrosis factor receptor superfamily, member 10b
TNFRSF10C	NM_003841.2	tumor necrosis factor receptor superfamily, member 10c, decoy without an intracellular domain
TNFRSF10D	NM_003840.3	tumor necrosis factor receptor superfamily, member 10d, decoy with truncated death domain
TNFSF10	NM_003810.2	tumor necrosis factor (ligand) superfamily, member 10
TNN	NM_022093.1	tenascin N
TNR	NM_003285.2	tenascin R
TP53	NM_000546.2	tumor protein p53
TPO	NM_175722.1	thyroid peroxidase
TRAF7	NM_032271.2	TNF receptor-associated factor 7, E3 ubiquitin protein ligase
TSC1	NM_000368.3	tuberous sclerosis 1
TSHR	NM_001018036.2	thyroid stimulating hormone receptor
TSLP	NM_033035.4	thymic stromal lymphopoietin
TSPAN7	NM_004615.3	tetraspanin 7
TTK	NM_003318.3	TTK protein kinase
U2AF1	NM_001025203.1	U2 small nuclear RNA auxiliary factor 1
UBB	NM_018955.2	ubiquitin B
UBE2T	NM_014176.3	ubiquitin-conjugating enzyme E2T (putative)
UTY	NM_007125.3	ubiquitously transcribed tetratricopeptide repeat containing, Y-linked
VEGFA	NM_001025366.1	vascular endothelial growth factor A
VEGFC	NM_005429.2	vascular endothelial growth factor C
VHL	NM_000551.2	von Hippel-Lindau tumor suppressor, E3 ubiquitin protein ligase
WEE1	NM_003390.3	WEE1 G2 checkpoint kinase
WHSC1	NM_007331.1	Wolf-Hirschhorn syndrome candidate 1
WHSC1L1	NM_017778.2	Wolf-Hirschhorn syndrome candidate 1-like 1
WIF1	NM_007191.2	WNT inhibitory factor 1
WNT10A	NM_025216.2	wingless-type MMTV integration site family, member 10A
WNT10B	NM_003394.2	wingless-type MMTV integration site family, member 10B
WNT11	NM_004626.2	wingless-type MMTV integration site family, member 11
WNT16	NM_057168.1	wingless-type MMTV integration site family, member 16
WNT2	NM_003391.2	wingless-type MMTV integration site family member 2

WNT2B	NM_024494.1	wingless-type MMTV integration site family, member 2B
WNT3	NM_030753.3	wingless-type MMTV integration site family, member 3
WNT4	NM_030761.3	wingless-type MMTV integration site family, member 4
WNT5A	NM_003392.3	wingless-type MMTV integration site family, member 5A
WNT5B	NM_032642.2	wingless-type MMTV integration site family, member 5B
WNT6	NM_006522.3	wingless-type MMTV integration site family, member 6
WNT7A	NM_004625.3	wingless-type MMTV integration site family, member 7A
WNT7B	NM_058238.1	wingless-type MMTV integration site family, member 7B
WT1	NM_000378.3	Wilms tumor 1
XPA	NM_000380.3	xeroderma pigmentosum, complementation group A
XRCC4	NM_003401.3	X-ray repair complementing defective repair in Chinese hamster cells 4
ZAK	NM_016653.2	sterile alpha motif and leucine zipper containing kinase AZK
ZBTB16	NM_006006.4	zinc finger and BTB domain containing 16
ZBTB32	NM_014383.1	zinc finger and BTB domain containing 32
ZIC2	NM_007129.2	Zic family member 2

Table S2: List of investigated proteins

Coordinate	Target/Control	Coodinate	Target/Control
A1, A2	Reference Spots	C13, C14	HO-2/HMOX2
A23, A24	Reference Spots	C15, C16	HSP27
B1, B2	Bad	C17, C18	HSP60
B3, B4	Bax	C19, C20	HSP70
B5, B6	Bcl-2	C21, C22	HTRA2/Omi
B7, B8	Bcl-x	C23, C24	Livin
B9, B10	Pro-Caspase-3	D1, D2	PON2
B11, B12	Cleaved Caspase-3	D3, D4	p21/CIP1/CDKN1A
B13, B14	Catalase	D5, D6	p27/Kip1
B15, B16	cIAP-1	D7, D8	Phospho-p53 (S15)
B17, B18	cIAP-2	D9, D10	Phospho-p53 (S46)
B19, B20	Claspin	D11, D12	Phospho-p53 (S392)
B21, B22	Clusterin	D13, D14	Phospho-Rad17 (S635)
B23, B24	Cytochrome c	D15, D16	SMAC/Diablo
C1, C2	TRAIL R1/DR4	D17, D18	Survivin
C3, C4	TRAIL R2/DR5	D19, D20	TNF RI/TNFRSF1A
C5, C6	FADD	D21, D22	XIAP
C7, C8	Fas/TNFRSF6/CD95	D23, D24	PBS (Negative Control)
C9, C10	HIF-1α	E1, E2	Reference Spots
C11, C12	HO-1/HMOX1/HSP32		

Figure S1: Volcano plot displays log10 (p-value) and log2 fold change for each gene -with the selected covariate. Highly statistically significant genes fall at the top of the plot, and highly differentially expressed genes fall to either side. Green point colors and horizontal lines indicate various False Discovery Rate (FDR) thresholds.

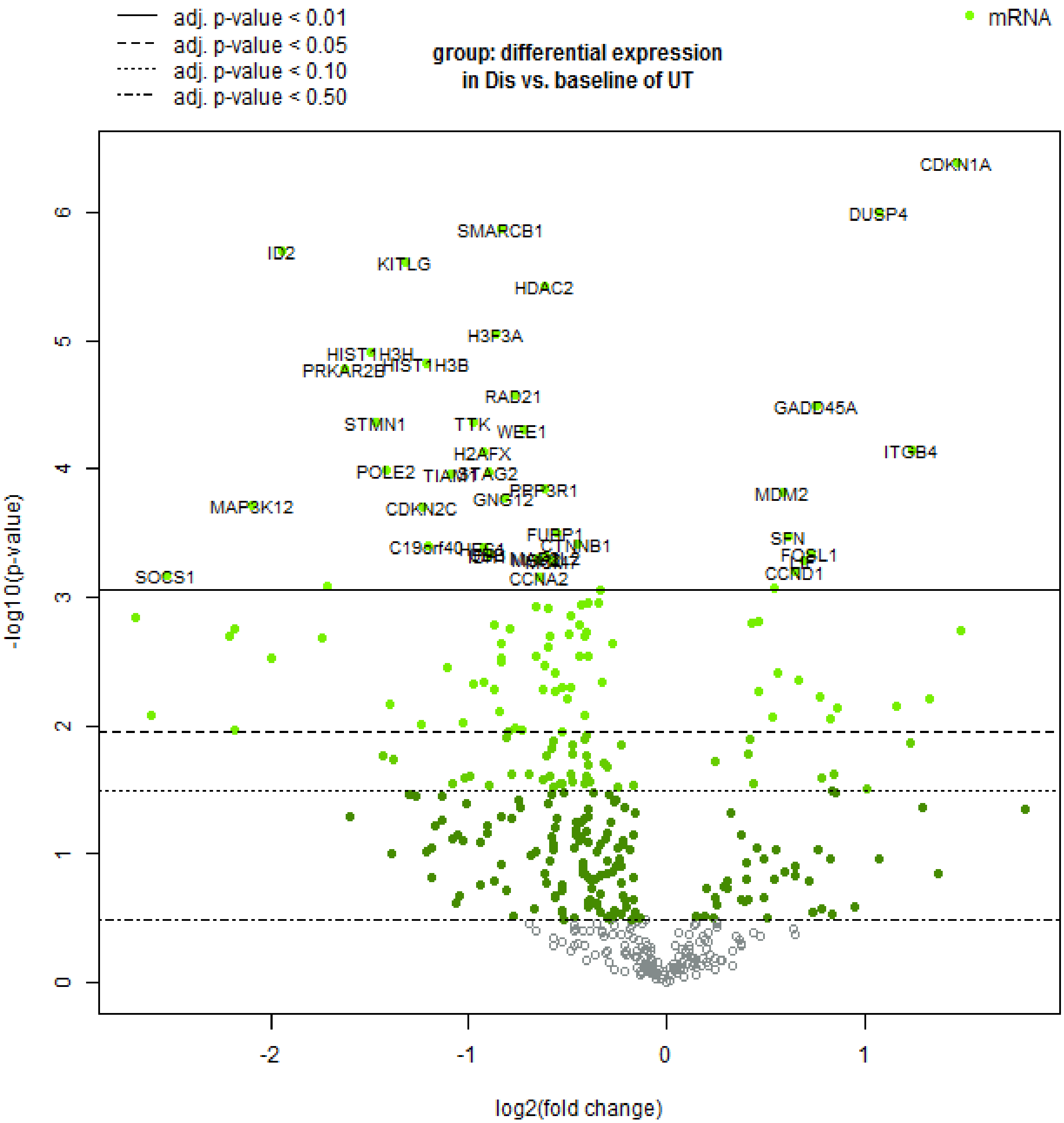

Supplement: Supplementary file 1 — Dataset [file 41598_2019_41685_MOESM1_ESM.pdf]
